# The Implication of Early Chromatin Changes in X Chromosome Inactivation

**DOI:** 10.1016/j.cell.2018.11.041

**Published:** 2019-01-10

**Authors:** Jan Jakub Żylicz, Aurélie Bousard, Kristina Žumer, Francois Dossin, Eusra Mohammad, Simão Teixeira da Rocha, Björn Schwalb, Laurène Syx, Florent Dingli, Damarys Loew, Patrick Cramer, Edith Heard

**Affiliations:** 1 Institut Curie, PSL Research University, CNRS UMR3215, INSERM U934, UPMC Paris-Sorbonne, 75005 Paris, France; 2 University of Cambridge, Department of Physiology, Development and Neuroscience, Cambridge CB2 3EG, UK; 3 Max Planck Institute for Biophysical Chemistry, Department of Molecular Biology, 37077 Göttingen, Germany; 4 Instituto de Medicina Molecular, Faculdade de Medicina, Universidade de Lisboa, 1649-028 Lisboa, Portugal; 5 Institut Curie, PSL Research University, Centre de Recherche, Laboratoire de Spectrométrie de Masse Protéomique, Paris 75248 Cedex 05, France

**Keywords:** epigenetics, X chromosome inactivation, embryonic stem cells, Xist, histone deacetylase, histone acetylation, Polycomb, PRC1, PRC2

## Abstract

During development, the precise relationships between transcription and chromatin modifications often remain unclear. We use the X chromosome inactivation (XCI) paradigm to explore the implication of chromatin changes in gene silencing. Using female mouse embryonic stem cells, we initiate XCI by inducing *Xist* and then monitor the temporal changes in transcription and chromatin by allele-specific profiling. This reveals histone deacetylation and H2AK119 ubiquitination as the earliest chromatin alterations during XCI. We show that HDAC3 is pre-bound on the X chromosome and that, upon *Xist* coating, its activity is required for efficient gene silencing. We also reveal that first PRC1-associated H2AK119Ub and then PRC2-associated H3K27me3 accumulate initially at large intergenic domains that can then spread into genes only in the context of histone deacetylation and gene silencing. Our results reveal the hierarchy of chromatin events during the initiation of XCI and identify key roles for chromatin in the early steps of transcriptional silencing.

## Highlights

• An epigenomic roadmap for initiation of X chromosome inactivation (XCI)
• Histone deacetylation and H2A ubiquitination are among the earliest XCI events
• HDAC3-mediated histone deacetylation is required for efficient XCI
• PcG marks are first deposited intergenically and spread when gene silencing occurs

## Graphical Abstract

**Figure:**
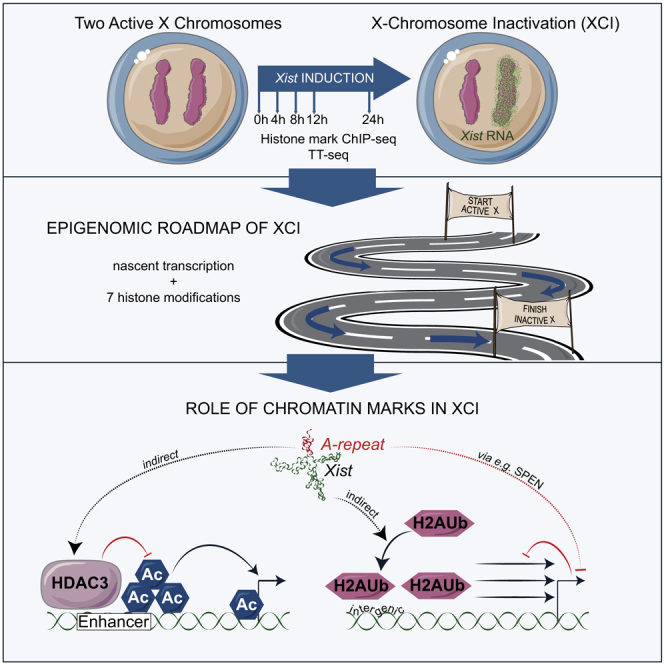

## Teaser

Żylicz et al. provide a detailed characterization of the earliest stages of X chromosome inactivation, tracing chromatin modification dynamics and uncovering the key role of chromatin changes in initiation of gene silencing.

## Introduction

Successful development requires the establishment and maintenance of euchromatin and heterochromatin in different parts of the genome. Progressively stable silencing of some genic regions occurs as epigenetic memory continues to accrue, finally leading to facultative heterochromatin formation (Trojer and Reinberg, 2007). Multiple layers of chromatin modifications are believed to enable stable transcriptional silencing. However, little is known about how facultative heterochromatin is dynamically formed and to what extent chromatin changes are involved in the establishment of gene silencing. A powerful model for developmentally induced gene silencing and formation of facultative heterochromatin is X chromosome inactivation (XCI) in female mammals. Although the role for chromatin changes in maintenance of the inactive state has been extensively studied (Disteche and Berletch, 2015), almost nothing is known about their role in the initiation of gene silencing.

In female mouse embryos, one of the two X chromosomes is randomly chosen for inactivation around the time of implantation (Lyon, 1962). This phenomenon is dependent on the coating of the future inactive X chromosome (Xi) by the long non-coding RNA *Xist* (Penny et al., 1996). The conserved A-repeat region of *Xist* mediates transcriptional silencing (Wutz et al., 2002), whereas other parts of *Xist* ensure chromosome coating (Almeida et al., 2017, Wutz et al., 2002). Assays coupling immunofluorescence with RNA fluorescence *in situ* hybridization (IF/RNA FISH) have revealed that, upon *Xist* RNA coating, a program of striking chromatin rearrangements ensues. These include rapid loss of histone modifications associated with active promoters (H3K4me3, H4ac, H3K9ac, and H3K27ac) and enhancers (H3K27ac and H3K4me1) (Chaumeil et al., 2002, Jeppesen and Turner, 1993). Furthermore, there is accumulation of H2AK119Ub and H3K27me3, two repressive histone marks dependent on the activity of Polycomb repressive complex (PRC) 1 and 2, respectively (for a review, see Brockdorff, 2017). These progressive alterations of chromatin states are associated with stable repression of the majority of genes on the Xi. Not all X-linked loci are affected in the same way, however, with some genes being silenced much faster than others or even entirely resisting repression (“escapees”) (Disteche and Berletch, 2015). The reason for this striking diversity in gene inactivation dynamics remains unclear. It is likely that the susceptibility of loci to *Xist* spreading plays a role in this process (Borensztein et al., 2017, Engreitz et al., 2013); however, differences in their chromatin status could also underpin transcriptional silencing dynamics.

Although some chromatin marks have previously been mapped on the X chromosome in female embryonic stem cells (ESCs) and differentiated cells, nothing is known about the dynamics of the XCI process, especially during early stages when gene silencing actually takes place (Marks et al., 2009, Pinter et al., 2012). Specifically, the order of chromatin changes and their possible role(s) in mediating transcriptional silencing remain largely unknown. Previous studies have relied on ESC differentiation for XCI initiation, which results in highly asynchronous *Xist* induction and elevated levels of heterogeneity in the population. These complications entirely mask primary chromatin events occurring in a small subset of cells embarking on the process of XCI. Recent advances in the identification of *Xist* binding proteins revealed that most histone-modifying activities are not directly recruited by *Xist* RNA but, rather, by its binding partners, like SPEN and HNRNPK (Chu et al., 2015, McHugh et al., 2015, Monfort et al., 2015, Pintacuda et al., 2017). A major question is whether the chromatin changes that occur early on in XCI are actually involved in gene silencing during XCI and, if so, how.

Here we investigate the earliest events accompanying transcriptional silencing of the X chromosome. Using a female ESC line in which one X chromosome can be specifically inactivated via an inducible *Xist* gene, we performed allele-specific native chromatin immunoprecipitation sequencing (ChIP-seq) as well as nascent transcript profiling at the initiation stages of XCI. Using 4-hr time resolution, we were able to reveal the precise order of chromosome-wide epigenetic events that are intricately coupled to gene repression during XCI. We find that loss of histone acetylation and, in particular H3K27ac, is one of the first events following *Xist* RNA accumulation during initiation of XCI. We also show that histone deacetylation, via HDAC3, is vital for efficient silencing of most genes on the *Xist-*coated X chromosome. Contrary to the prevailing hypothesis, HDAC3 is not acutely recruited to the X chromosome by *Xist*; rather, it is pre-loaded, mainly at putative enhancers. Upon *Xist* coating, pre-loaded HDAC3 likely mediates histone deacetylation and facilitates transcriptional silencing. We also uncover a surprisingly rapid accumulation of the PRC1-dependent H2AK119Ub mark on the X chromosome, particularly at intergenic regions lying in proximity to *Xist* RNA entry sites, which are pre-marked by Polycomb (PcG) marks. Accumulation of H3K27me3 appears slightly later and is delayed compared with gene silencing. Using *Hdac3* and *Xist* mutant cells, we also show that spread of Polycomb marks into gene bodies can only occur when transcriptional repression begins. Our study reveals the chromatin choreography across the X chromosome during the early steps of XCI initiation and uncovers a role for histone deacetylation in the establishment of gene silencing.

## Results

### Allele-Specific Native ChIP-Seq Monitors Chromatin Changes during XCI

**Figure 1:**
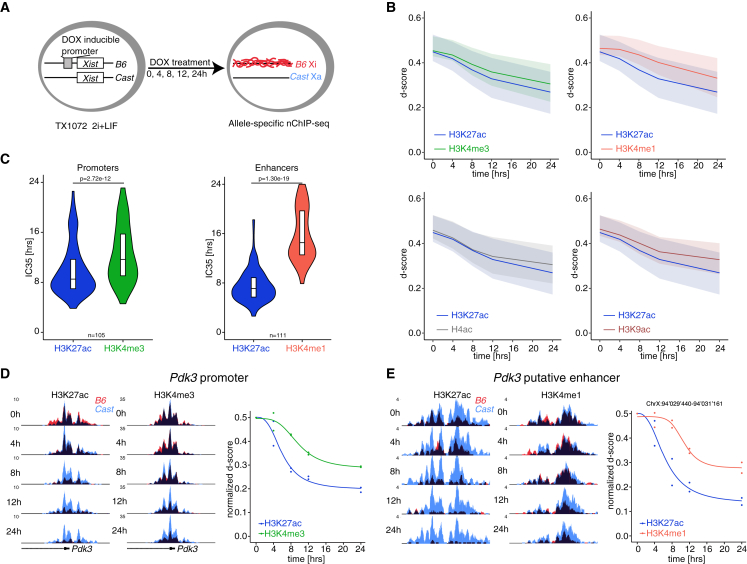
Histone Deacetylation Is among the First Events of XCI (A) Schematic representation of the experimental design. The hybrid TX1072 mouse ESC line was used, in which *Xist* can be induced from the endogenous B6 allele. (B) H3K27ac (blue) deacetylation dynamics in comparison with the loss of other active histone marks. Shown are average d-scores (shading is the interquartile range) of all peaks at the X chromosome. (C) Paired comparison of the dynamics in H3K27ac and H3K4 methylation loss. Plotted are IC35 parameters of peaks at active promoters (left) and putative active enhancers (right) and within the quantitative range of the experiment (IC35 < 24 hr). The p value is from a paired Wilcoxon rank-sum test. (D and E) Genome browser tracks showing H3K27ac and H3K4me3 at the *Pdk3* promoter (D) or H3K27ac and H3K4me1 at a putative *Pdk3* enhancer (E). Allele-specific tracks are overlaid (Cast reads in blue; B6 reads in red). The plots show the quantification of the inactivation dynamics with fitted sigmoidal curves. See also Figure S1.

**Figure S1:**
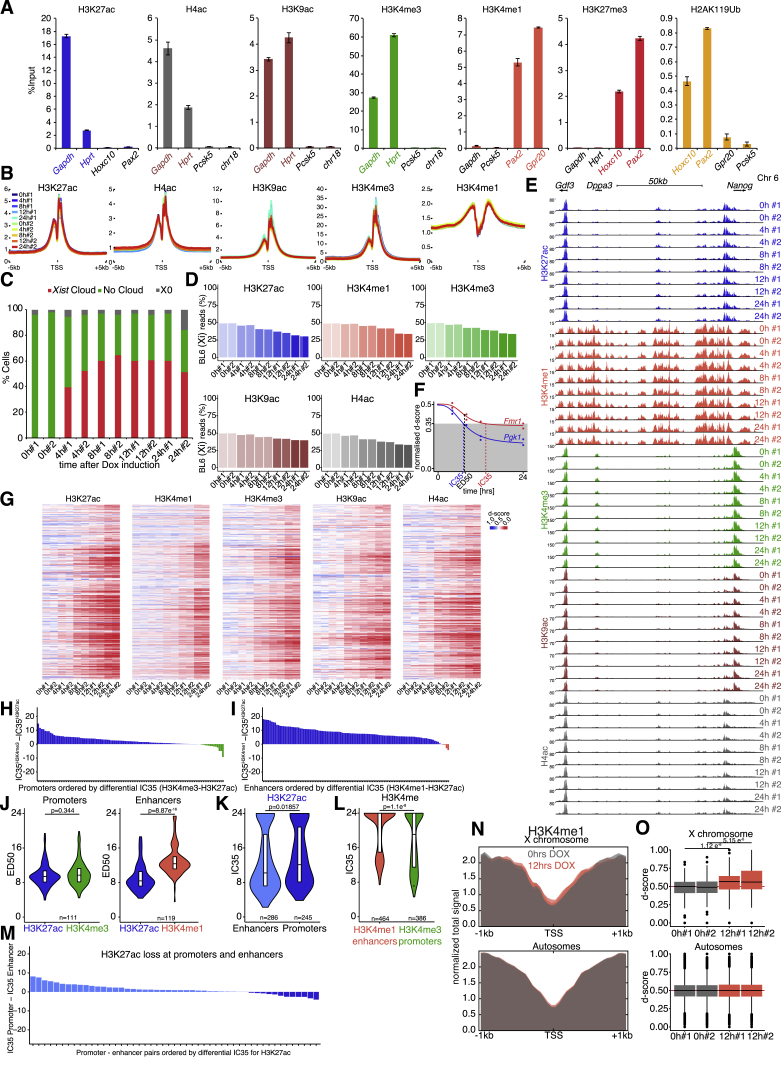
Allele-Specific Native ChIP-Seq Monitors Changes in Active Histone Modifications during XCI, Related to Figure 1 (A) nChIP enrichment validation using qPCR. Shown is the enrichment of nChIP signal at positive (color) and negative (black) control regions for each mark individually. Shown is a representative result for one biological replicate. Values were normalized to the input sample. (B) Average enrichment plots for active histone modifications across all transcriptional start sites. (C) RNA FISH quantification of *Xist* induction (p510 probe) during both replicates of nChIP-seq time course. At least 70 nuclei were quantified for each sample. (D) Bar plots showing the percentage of B6(Xi) reads mapping to the X chromosome in the nChIP-seq time course. (E) Genome browser plots of active histone modification at the *Gda3/Dppa3/Nanog* cluster. Shown are all mapped reads at all time points in biological duplicates. Note highly reproducible and stable pattern of enrichment. (F) Example showcasing the differences between IC35 and ED_50_ parameters. The IC35 measures when the loss of a histone mark reaches an efficiency threshold and not the timing when this process occurs most rapidly. For example, if two marks show different efficiency of loss from the Xi (i.e., different IC35) the process might still be occurring at the same time. Shown is H3K27 deacetylation dynamics for two promoters (*Fmr1*:red, *Pgk1*:blue). Both promoters have the same ED_50_ (i.e., time when sigmoid reaches its maximum slope), however show very different efficiency of deacetylation. The latter difference is captured by the IC35 parameter (i.e., time when sigmoid reaches the 0.35 threshold). (G) Heatmaps showing the d-scores of all peaks on the X chromosome. Windows were sorted from centromere (top) to telomere (bottom). (H-I) Plots showing the distribution of differential IC35 (H): H3K4me3-H3K27ac; (I): H3K4me1-H3K27ac) for all promoters (H) and putative enhancers (I). Each bar represents a single window, these were ordered accordingly to their differential IC35. (J) Violin plots comparing the dynamics of H3K27ac and H3K4 methylation loss at active promoters (left) and putative active enhancers (right). Plots compare the ED_50_ parameter of H3K27ac and H3K4me peaks within the quantitative range of the experiment (IC35 < 24hrs). p value was calculated using paired Wilcoxon rank sum test.(K-L) Violin plots comparing the distribution of IC35 for H3K27ac (K) or H3K4me1/3 (L) at enhancers and promoters. p values calculated using Wilcoxon rank sum test. (M) Plots showing the distribution of differential IC35 for H3K27ac at promoter-putative enhancer pairs. Windows within one TAD were paired based on proximity. Individual pairs were ordered accordingly to their differential IC35. (N) Average H3K4me1 distribution plots around the transcriptional start site of active genes (TSS ± 1kb). Plots were done using all mapped reads on TSS of active genes on X chromosome (top) and on autosomes (bottom). Average profiles show duplicates at 0hr (gray) and 12hrs (orange) of DOX treatment. (O) Boxplots showing the d-score distribution for H3K4me1 around TSS of active genes (±200bp) after 0 (gray) and 12hrs (orange) of DOX treatment, on X chromosome (top) and on autosomes (bottom). p values calculated using Wilcoxon rank sum test.

A molecular roadmap of the earliest chromatin changes during the initiation of XCI has not yet been established. To reduce heterogeneity and obtain a highly resolved time series of chromatin events upon *Xist* RNA coating, we used the TX1072 ESC line (Figure 1A; Schulz et al., 2014). This hybrid (*Mus musculus castaneus* x C57BL/6) line harbors a doxycycline (DOX) inducible promoter upstream of the endogenous *Xist* at the C57BL/6 (B6) allele (Schulz et al., 2014, Wutz et al., 2002). By adding DOX, we synchronously induce *Xist* expression and can thus decouple the onset of XCI from differentiation-dependent chromatin changes (Figure S1C). We performed allele-specific native ChIP-seq (nChIP-seq) for seven histone modifications across five time points at up to 4-hr resolution on biological duplicates (Figure 1A; Figures S1A and S1B). We focused on histone modifications associated with active promoters (H3K4me3, H3K9ac, H4ac, and H3K27ac) and active enhancers (H3K4me1 and H3K27ac) as well as repressive Polycomb-dependent marks (H3K27me3 and H2AK119Ub). All nChIP-seq datasets were sequenced to a depth of at least 40 million reads and showed a high signal-to-noise ratio (Figures S1B and S1E). The reads were split according to content of allele-specific SNPs (*B6*, mapping to Xi; *Cast*, mapping to active X); this allelic information was then analyzed in detail to define XCI-specific changes.

### Histone Deacetylation Is One of the Earliest Events during Initiation of XCI

Upon *Xist* coating, the Xi becomes rapidly depleted of active histone modifications. Although this global loss of euchromatin occurs in a similar time window as the changes in X-linked transcriptional activity (Chaumeil et al., 2002), the precise chronology and possible mechanistic relationship of these two events have remained unclear. To address this, we first analyzed nChIP-seq datasets for a panel of active histone modifications (H3K27ac, H3K4me1, H3K4me3, H3K9ac, and H4ac; Figure S1E). This revealed progressive depletion of B6-specific reads (originating from Xi) upon *Xist* induction, with different marks showing different kinetics of loss (Figure S1D). To analyze this in detail, we performed peak calling on all the reads (STAR Methods). For each peak with at least 50 allele-specific reads, we calculated its d-score (reads^B6^/[reads^Cast^ + reads^B6^]). This parameter of allelic skewing uses the active X chromosome as a powerful internal control, rendering the analysis less sensitive to variability in nChIP-seq quality and, thus, more stable between replicates.

We first set out to track the relative dynamics of active chromatin marks by comparing d-score dynamics of all peaks. We found that loss of H3K27ac and H4ac is among the earliest and most robust chromatin events on the X chromosome following *Xist* induction (Figure 1B). Furthermore, we found that not only did different histone modifications show distinct behaviors but, also, that specific regions of the X chromosome followed rather different dynamics of inactivation (Figure S1G). Thus, there is a temporal hierarchy of chromatin events both at the level of different histone marks and genomic loci.

To better understand in which context chromatin changes might affect gene silencing, we separately focused on promoters and putative enhancers. For each window, we plotted a d-score (normalized to t = 0 hr) as a function of time and fitted a sigmoidal curve (STAR Methods). We found that the maximal dynamic range of inactivation was from 0.5 to ∼0.2 and, thus, set a threshold for when the curve reached 50% of this dynamic range; i.e., d = 0.35 (STAR Methods). This IC35 (inactivation criterium 0.35) parameter indicates how quickly a given peak becomes significantly decreased or lost (Figure S1F). Pairwise comparisons of the IC35 at X-linked gene promoters revealed that H3K27ac becomes efficiently deacetylated prior to demethylation of H3K4me3 (Figure 1C). This trend holds true for ∼88% of measured promoters (Figure S1H), including a representative example of the *Pdk3* promoter (Figure 1D). Similar analysis of the enhancer inactivation dynamics revealed that H3K27ac is efficiently depleted prior to loss of H3K4me1 (Figure 1C). This robust difference is observed in ∼95% of the cases; e.g., at a putative *Pdk3* enhancer element (Figure 1E; Figure S1I). Thus, histone deacetylation is an early and efficient event during XCI.

Next, to extract information about relative timing rather than the efficiency threshold, we obtained the time when the curve reaches its maximum slope (effective dose 50%, ED_50_; Figure S1F). ED_50_ analysis revealed that both H3K27ac and H3K4me3 become depleted concomitantly (Figure S1J). Together with the IC35 data, this indicates that, even though demethylation of promoters and their deacetylation are concurrent, the latter process is significantly more efficient. Furthermore, unlike promoters, enhancers showed significant differences in the ED_50_ parameter between H3K27ac and H3K4me1, indicating that enhancer demethylation is not only less efficient but also significantly delayed (Figure S1J).

To assess the relative deacetylation dynamics of enhancers and promoters, we compared the IC35 parameter for H3K27ac at both types of genomic regulatory elements. Intriguingly, enhancer deacetylation was slightly more efficient than that of promoters (Figure S1K). After pairing active promoters to their putative enhancers (STAR Methods), ∼76% (37 of 49) of them showed more efficient deacetylation at enhancers than at promoters (Figure S1M). On the other hand, loss of H3K4me1 on the Xi, which is indicative of enhancer decommissioning, was found to be significantly less efficient compared with the loss of H3K4me3 at promoters (Figure S1L). Thus, inactivation (loss of H3K27ac) of enhancers proceeds at a slightly higher efficiency than of promoters, although stable decommissioning (loss of H3K4me) of promoters precedes that of enhancers. Intriguingly, upon H3K4me3 loss following *Xist* induction, we observe a slight increase in H3K4me1 levels around transcriptional start sites (TSSs), which probably reflects an intermediate of stepwise enzymatic H3K4me3 demethylation (Figures S1N and S1O).

In summary, our data show that efficient histone deacetylation at promoters and enhancers is one of the first events during XCI, indicating that it might contribute to initiation of transcriptional silencing. In comparison, demethylation of H3K4me3 and H3K4me1 is less efficient and delayed, respectively. Finally, deacetylation of promoters and enhancers seems to follow broadly comparable dynamics, whereas their decommissioning differs significantly.

### Histone Deacetylation Is Tightly Linked to Transcriptional Silencing

**Figure 2:**
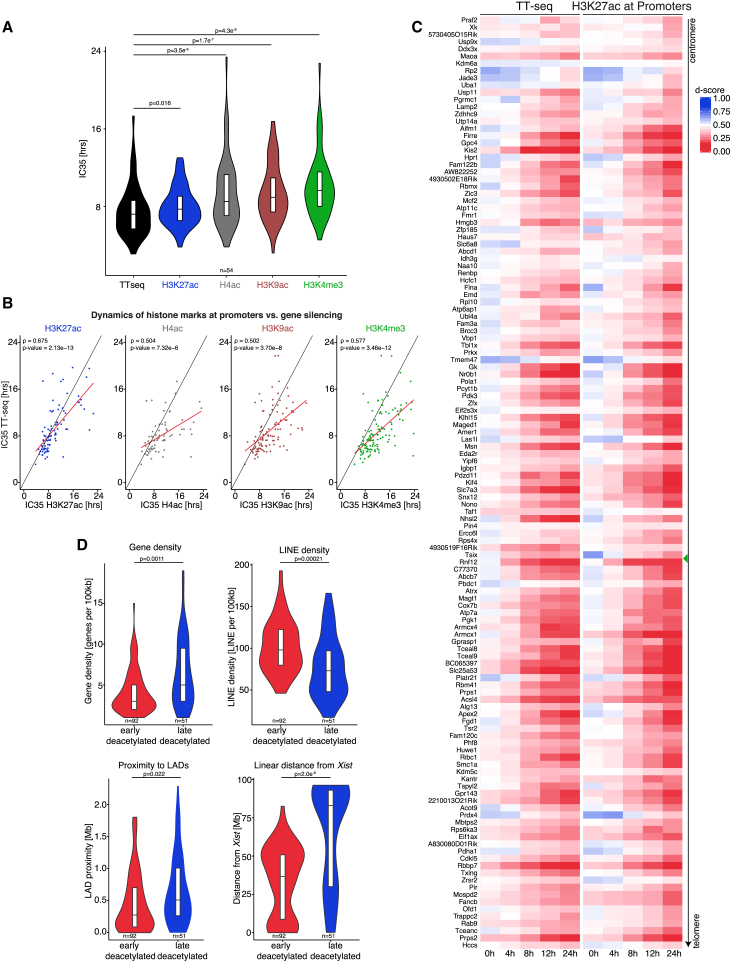
Histone Deacetylation Is Tightly Correlated with Transcriptional Silencing (A) Comparison of IC35 parameters for gene silencing (TT-seq) and histone modifications at associated promoters. The p values are from a paired Wilcoxon rank-sum test. (B) Pairwise comparison of IC35 from gene silencing and histone mark dynamics as in (A). Linear regression fitting (red) and perfect correlation with slope = 1 (black) is shown. Pearson’s correlation (ρ) and p value are shown for each set. (C) Heatmap showing gene silencing and associated promoter H3K27 deacetylation dynamics. Rows were ordered by their genomic position from centromere to telomere (the green triangle indicates the *Xist* location). The averages from biological duplicates are shown. (D) Quantification of features associated with early and late deacetylated promoters (H3K27ac). Shown are gene density, LINE density, proximity to LADs, and distance from the *Xist* locus. The p values are from a Wilcoxon rank-sum test with Benjamini Hochberg correction. See also Figure S2.

**Figure S2:**
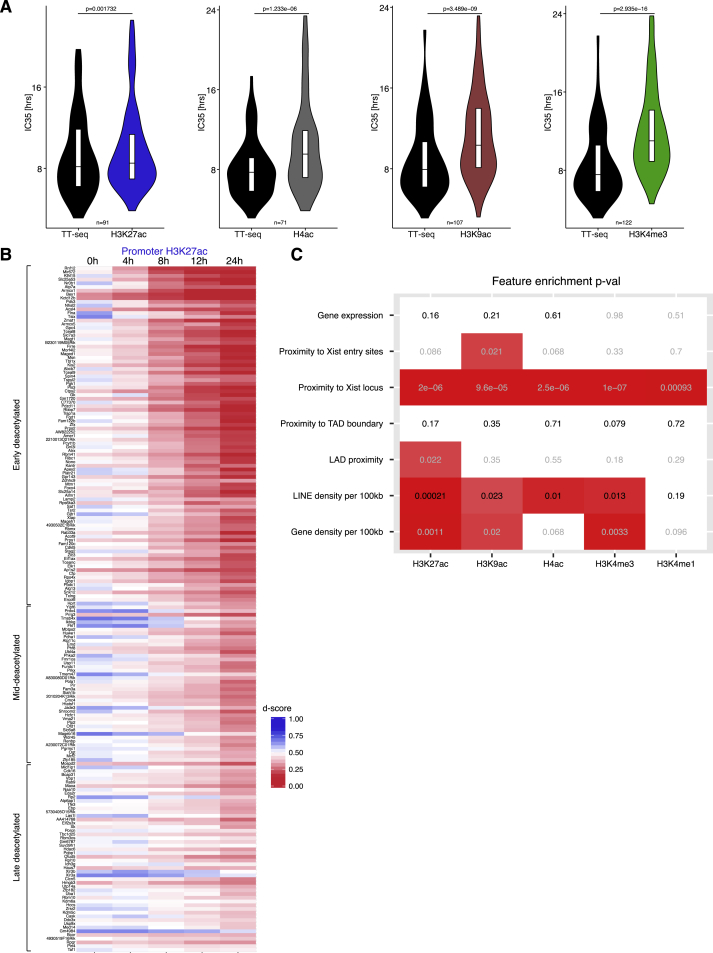
Histone Deacetylation Correlates with Transcriptional Silencing and Some Genomic Features, Related to Figure 2 (A) Violin plots comparing the dynamics of transcriptional silencing and the loss of active histone modifications from associated promoters. Plots compare the IC35 parameter calculated from TT-seq nascent transcription profiling to the IC35 of different active histone marks (H3K27ac, H4ac, H3K9ac, H3K4me3). p values were calculated using paired Wilcoxon rank sum test. (B) Heatmap of H3K27ac allelic dynamics at all promoters clustered using k-means. (C) An array showing features enriched (gray font) or depleted (black font) at promoters efficiently inactivated (low IC35) compared to late inactivated (higher IC35) using a panel of histone modifications. Shown are p values calculated using Wilcoxon rank sum test with Benjamini Hochberg correction. Red cells are reaching statistical significance of p < 0.05.

To gain insight into the relationship between loss of active chromatin marks and transcriptional silencing of X-linked genes, we compared our nChIP-seq dataset with transient transcriptome sequencing (TT-seq; Schwalb et al., 2016). This allows a direct measure of newly synthesized RNA and, thus, enabled us to measure transcriptional activity as the X chromosome becomes inactivated. Comparison of the IC35 for TT-seq and individual histone modifications at associated promoters revealed that transcriptional silencing dynamics are most tightly linked to promoter deacetylation at H3K27 and, to a lesser extent, to loss of H4ac, H3K9ac, and H3K4me3 (Figures 2A and 2B; Figure S2A). A similar pattern of gene silencing and promoter deacetylation dynamics further exemplifies this correlation (Figure 2C).

The striking variability in dynamics of promoter chromatin changes begged the question of whether these differences could be due to transcriptional status or genomic context; for example, proximity to *Xist* (the locus and/or *Xist* RNAs entry sites; Engreitz et al., 2013), gene density, or LINE (long interspersed nuclear elements) density. Indeed, the latter feature has been proposed previously to influence the efficiency of XCI (Chow et al., 2010, Loda et al., 2017; Figure S2B and S2C). We found that gene promoters that are rapidly H3K27-deacetylated preferentially reside in gene poor, LINE-dense regions in proximity to lamina-associated domains (LADs) and the *Xist* locus (Figure 2D).

Given that the histone deacetylation is the primary chromatin change observed upon *Xist* induction and that its kinetics are tightly linked to transcriptional silencing, we decided to investigate whether the loss of histone acetylation might play a functional role in mediating the initiation of gene silencing during XCI.

### HDAC3 Mediates Efficient Transcriptional Silencing during XCI

**Figure S3:**
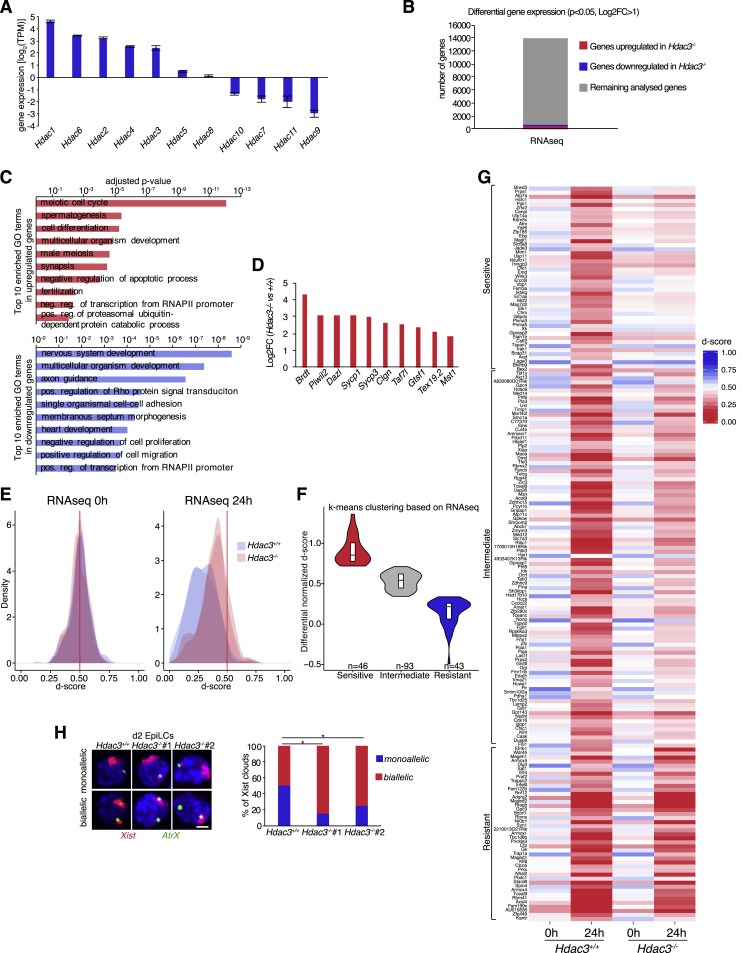
HDAC3 Mediates Efficient Trascriptional Silencing during XCI; RNA-Seq Analysis and Validation Related to Figure 3 (A) Expression level of all *Hdac* enzymes in TX1072 ESC. Shown is average from two biological replicates (+/− StDev). (B) Plot showing the number of differentially expressed genes in *Hdac3*^*−/−*^ cells. EdgeR p value < 0.05 and Log2FC > 1 (upregulated) or Log2FC < −1 (downregulated). Related to Table S1. (C) Top ten enriched Gene Ontology terms in upregulated (top) and downregulated genes (bottom) in *Hdac3*^*−/−*^ cells. (D) Examples of upregulated meiosis genes in *Hdac3*^*−/−*^ cells. Shown is average Log2FC from two biological replicates. Typically, these genes are stably repressed by DNA methylation, however here in a hypomethylated context of ESCs grown in 2i/LIF conditions, HDAC3 is responsible for suppressing their transcription. (E) Density plot representing allelic skewing of all X-linked genes after 0 and 24hrs of DOX treatment in *Hdac3*^*+/+*^ and *Hdac3*^*−/−*^ lines. Data was extracted from RNA-seq results and presented is the distribution of d-scores, shown are duplicates of the control ESC line and two independent *Hdac3* knockout clones. Red line shows d-score = 0.5 equating to biallelic expression. (F) Violin plot showing the distribution of normalized differential d-scores calculated from RNA-seq experiments in *Hdac3*^*+/+*^ and *Hdac3*^*−/−*^ ESCs. Genes were clustered using k-means. The gene clusters are presented in a heatmap of average d-scores (G). (H) Validation of *Hdac3*^*−/−*^ phenotype in day 2 EpiLCs induced without DOX. FISH was performed with probes for *Xist* (p510-Red) and an X-linked gene *AtrX* (Green). At least 100 nuclei with *Xist* clouds were imaged to assess the *AtrX* silencing status. ^∗^Chi2 p value < 0.05. Scale bar = 5 μm.

**Figure 3:**
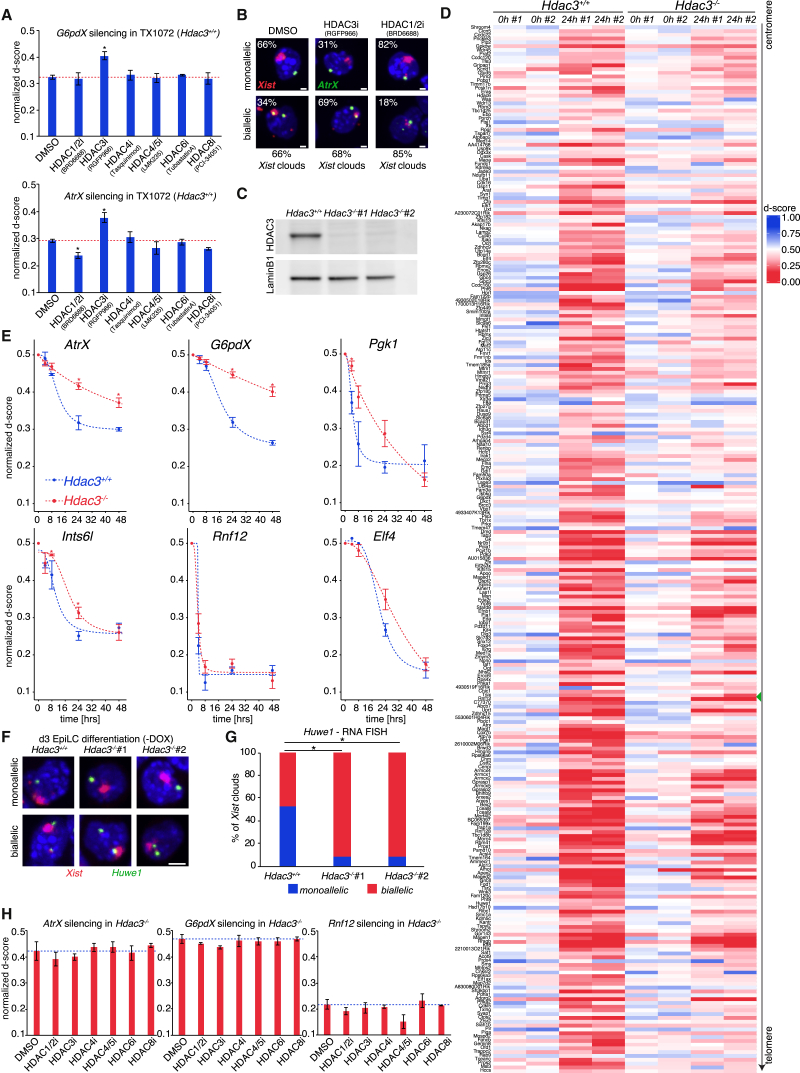
HDAC3 Mediates Efficient Transcriptional Silencing during XCI (A) Screening for HDAC activity involved in XCI using selective inhibitors and pyrosequencing for *G6pdX* and *AtrX*. The red line represents silencing in the DMSO control after 24 hr of DOX treatment. Shown are averages (± SD) from 2 experiments. ^∗^p < 0.05, Student’s t test. (B) RNA FISH for *Xist* (red) and the X-linked *AtrX* gene (green) on ESCs treated as in (A). Percentages of biallelic and monoallelic *AtrX* expression in *Xist*-expressing cells are shown (>100 nuclei quantified, scale bar, 10 μm). The quantification of *Xist* cloud formation is shown below. (C) Immunoblot analysis of whole-cell lysates from TX1072 (*Hdac3*^*+/+*^) and two *Hdac3*^*−/−*^ clones probed with antibodies specific for the N terminus of HDAC3 and Lamin B1. (D) Heatmap showing gene silencing after 0 and 24 hr of DOX treatment in TX1072 (*Hdac3*^*+/+*^) and two *Hdac3*^*−/−*^ clones. Rows were ordered by their genomic position from centromere to telomere (the green triangle indicates the *Xist* location). (E) RNA-seq validation using pyrosequencing on ESCs (TX1072: *Hdac3*^*+/+*^ and *Hdac3*^*−/−*^) differentiated to EpiLCs with DOX. Shown are averages (± SEM) from 3 independent experiments and two *Hdac3*^*−/−*^ clones for 3 genes sensitive to HDAC3 loss (top) and 3 genes less dependent on its activity (bottom). ^∗^p < 0.05, Student’s t test. (F and G) Validation of *Hdac3*^*−/−*^ phenotype in day 3 EpiLCs differentiated without DOX. (F) FISH was performed with probes for *Xist* (p510, red) and an X-linked gene, *Huwe1* (green). Scale bar, 5 μm. (G) Also shown is quantification of *Huwe1* silencing status in more than 100 nuclei with *Xist* clouds. χ^2^ test, ^∗^p < 0.05. (H) Screening for HDAC activity compensating for HDAC3 loss using pyrosequencing for *AtrX*, *G6pdX*, and *Rnf12*. The experiment was performed like in (A) but in *Hdac3*^*−/−*^ cells. Shown are averages (± SD) from two independent clones. ^∗^p < 0.05, Student’s t test. See also Figure S3 and Table S1.

To investigate whether this rapid histone deacetylation is mediated by histone deacetylases (HDACs), we first tested selective pharmacological inhibitors against all HDACs expressed in ESCs (Figure S3A). Importantly, after 24 hr of DOX induction, only HDAC3 catalytic inhibition resulted in a significant silencing defect compared with the control (DMSO) (Figure 3A). We further validated these findings using nascent RNA FISH and, unexpectedly, found that HDAC1 and 2 inhibition promoted more efficient *Xist* upregulation, possibly because of rtTA upregulation (Figure 3B). The finding that HDAC3 is involved in XCI is in line with previous reports that it might interact with SPEN, which is one of the key *Xist*-silencing factors (Chu et al., 2015, McHugh et al., 2015, Monfort et al., 2015). Also, *Hdac3* knockdown was recently shown to result in defective silencing of another X-linked gene: *Gpc4* (McHugh et al., 2015).

To better interrogate the specific functions of HDAC3 in XCI, we generated two independent *Hdac3*^*−/−*^ TX1072 deletion clones using CRISPR/Cas9 technology (STAR Methods; Figure 3C). We performed RNA sequencing (RNA-seq) on these, both prior to and 24 hr following DOX addition. Differential gene expression analysis revealed relatively mild changes apart from some meiosis-associated genes (Figures S3B–S3D). Consistent with our inhibitor treatment results, allele-specific analysis revealed that both *Hdac3*^*−/−*^ clones are unable to efficiently silence X-linked genes (Figure S3E). Although the majority of genes across the X chromosome were affected, ∼24% of genes (43 of 182) were not, showing, instead, almost normal silencing (Figure 3D; Figures S3F and S3G). We further validated our findings in the context of EpiLC (epiblast like-cells) differentiation both with and without DOX treatment (Figures 3E–3G; Figure S3H). In our pyrosequencing experiments, we monitored the allelic bias in expression of genes affected (*AtrX*, *G6pdX*, and *Pgk1*) in their silencing by the HDAC3 loss and three less affected genes (*Ints6l*, *Rnf12*, and *Elf4*) (Figure 3E).

Our results indicate that loss of HDAC3 leads to a delay in transcriptional silencing of most X-linked genes. We noted that, over time, XCI is not fully prevented because gene silencing progressively ensues, although more slowly than in control cells (Figure 3E), indicating that mechanisms parallel to HDAC3 must also contribute to XCI. To address this further, we tested whether other HDACs might compensate for HDAC3 loss. To this end, we treated *Hdac3*^*−/−*^ ESCs with selective inhibitors against all expressed HDACs (1,2,3,4,5,6, and 8). We found that allelic skewing (i.e., XCI) of *AtrX*, *G6pdX*, and *Rnf12* were not affected in any of the treated cells (Figure 3H), arguing against the involvement of other HDACs in XCI. Thus, our data reveal that HDAC3 is the main histone deacetylase important for the initiation of silencing of most genes on the X chromosome. Because HDAC3 can mediate deacetylation of many substrates, we next investigated whether its effect on gene silencing is mediated by changes in chromatin status.

### HDAC3 Promotes Silencing by Rapidly Deacetylating Histones

**Figure 4:**
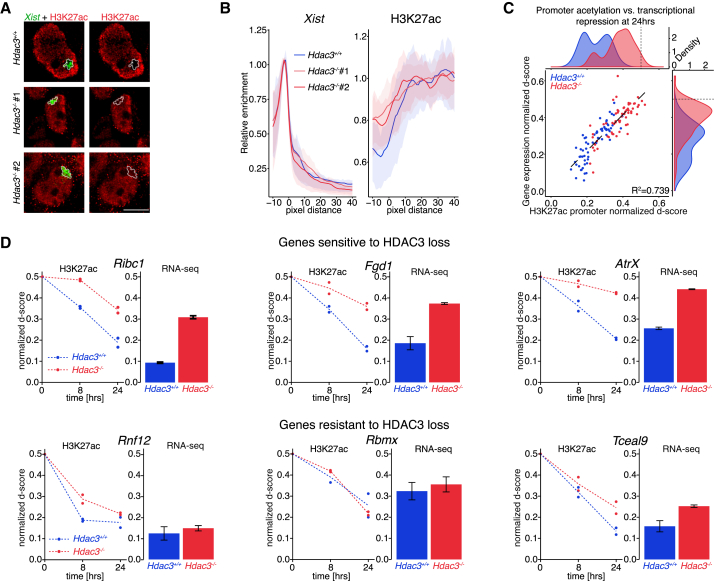
HDAC3 Promotes Silencing by Rapidly Deacetylating Histones (A) IF/RNA FISH on TX1072 (*Hdac3*^*+/+*^) and two *Hdac3*^*−/−*^ ESC clones induced with DOX for 24 hr. Cells were probed using anti-H3K27ac (red) antibodies and a *Xist* intronic probe (green). The white dotted line encircles the *Xist* domain. Because of technical variability, contrast settings were individually adjusted to reflect relative enrichment. Scale bar, 10 μm. (B) Quantification of (A) over at least 100 *Xist* clouds. Shown is the average signal for *Xist* and H3K27ac when profiles were aligned to the *Xist* cloud boundary (for more details, refer to Figure S4A and D). (C) Relationship between gene repression (RNA-seq) and promoter deacetylation (H3K27ac) at 24 hr in TX1072 (*Hdac3*^*+/+*^, blue) and *Hdac3*^*−/−*^ (red). On the scatterplot, the black dotted line represents fitted linear regression. ρ = 0.860. Density plots show the distribution of the normalized d-score for H3K27ac (horizontal) and RNA-seq (vertical); note that d-scores in mutant cells are shifted toward biallelic levels (0.5, dotted lines on density plots). Each dot represents the average normalized d-score from biological duplicates. (D) Comparison of promoter deacetylation dynamics and transcriptional silencing after 24 hr of DOX treatment in TX1072 (*Hdac3*^*+/+*^) and *Hdac3*^*−/−*^ ESCs. Examples of genes sensitive (top) and more resistant (bottom) to HDAC3 loss are shown. For RNA-seq, shown is the average from biological duplicates (± SD). See also Figure S4.

**Figure S4:**
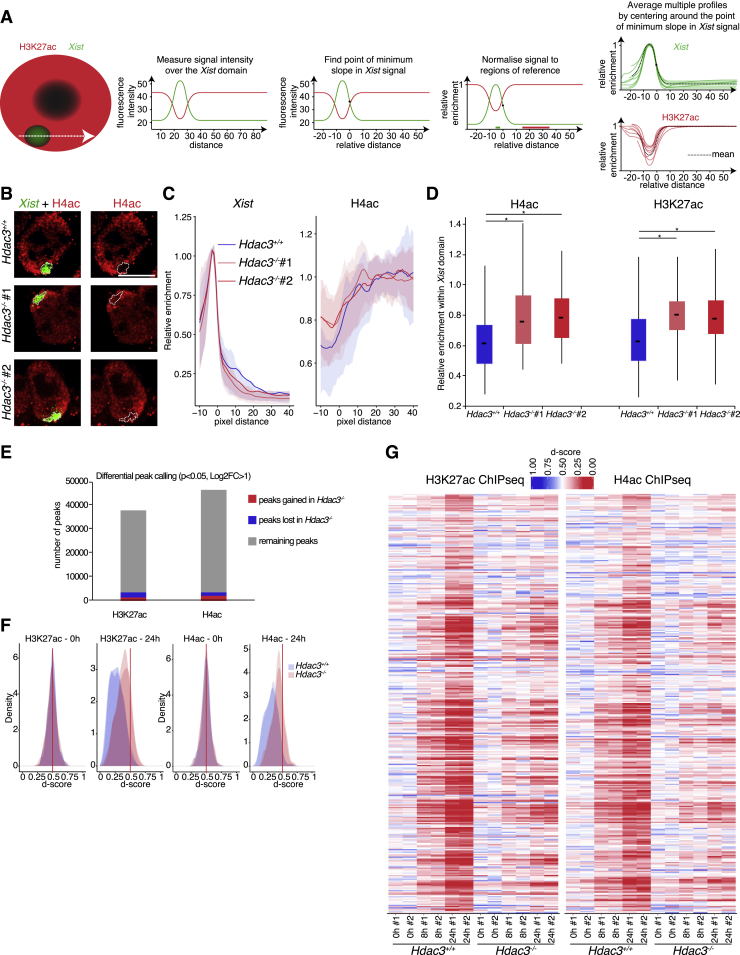
HDAC3 Promotes Silencing by Rapidly Deacetylating Histones; IF/FISH and nChIP-Seq Analysis, Related to Figure 4 (A) Schematics of IF/FISH quantification. For each nucleus with a *Xist* cloud a z-slice was selected where *Xist* signal reaches its maximum. A line was drawn across the *Xist* domain and into the nucleus avoiding the nucleolus region. Signal intensity for *Xist* (green) and IF (e.g., H3K27ac in red) was recorded along this axis. Next, *Xist* boundary was defined as a point of minimum slope of *Xist* signal. The X coordinates were centered around this boundary point. The fluorescence signal for each cell was normalized to reference regions of *Xist* peak (−4 to −2 pixels from the boundary) or IF signal plateau (15 to 35 pixels from the boundary), these regions are represented as color bars. Finally, profiles from multiple nuclei were averaged for each channel separately. (B) IF/RNA FISH on *Hdac3*^*+/+*^ and two *Hdac3*^*−/−*^ ESC clones induced with DOX for 24hrs. Cells were probed using anti-H4ac (red) antibodies and a *Xist* intronic probe (green). White dotted line encircles the *Xist* domain. Due to technical variability contrast settings were individually adjusted to reflect relative enrichment. Scale bar = 10 μm. (C) Quantification of IF/RNA FISH signal over at least 70 *Xist* clouds. Shown is the average signal for *Xist* and H4ac when profiles were aligned to the *Xist* cloud boundary. Signal was normalized per cell. For detailed explanation of IF/FISH quantification see point A.(D) RNA/FISH quantification based on point (C) and Figure 4A. Boxplots quantifying the relative enrichment of H4ac and H3K27ac within the *Xist* cloud (−10 to 0 pixel distance) in *Hdac3*^*+/+*^ and *Hdac3*^*−/−*^. *^∗^* p value < 0.05 from Wilcoxon rank sum test. (E) Plot showing the number of differentially called peaks in *Hdac3*^*−/−*^. P value < 0.05, Log2FC > 1 or Log2FC < −1. (F) Density plots showing the distribution of d-scores at H3K27ac (left) and H4ac (right) peaks on the X chromosome in *Hdac3*^*+/+*^ and *Hdac3*^*−/−*^. Initially, peaks are on average biallelic (0h) and become skewed toward the Cast allele after 24h of DOX treatment. Shown is an overlay of biological duplicates including two independent *Hdac3*^*−/−*^ clones. (G) Heatmaps showing the d-score evolution of all H3K27ac and H4ac peaks in *Hdac3*^*+/+*^ and *Hdac3*^*−/−*^. X-linked peaks were sorted based on their genomic position from the centromere to telomere.

Although our experiments using inhibitors and knockout ESCs indicated the importance of HDAC3 catalytic activity in mediating efficient XCI, it remained unclear whether histone deacetylation dynamics were also affected by loss of HDAC3. To address this question, we first monitored the global distribution of histone modifications by IF/RNA FISH. We observed that the Xi silent compartment formed much less efficiently in *Hdac3*^*−/−*^ ESCs, with increased levels of H3K27ac and H4ac remaining within the *Xist* cloud (Figures 4A and 4B; Figures S4A–S4D). To further explore the chromatin phenotype of *Hdac3*^*−/−*^ ESCs, we performed nChIP-seq for H3K27ac and H4ac at 0, 8, and 24 hr of DOX treatment. Differential peak analysis has revealed that less than 9% of peaks significantly change in *Hdac3*^*−/−*^ compared with WT cells at t = 0 hr (Figure S4E). Consistent with the IF/RNA FISH results, allele-specific analysis revealed delayed histone deacetylation in the *Hdac3*^*−/−*^ clones compared with the parental line (Figure 4C; Figures S4F and S4G). As with transcriptional silencing, the deacetylation defects affected most of the X chromosome but were not uniform, with some peaks being almost entirely resistant to HDAC3 loss (∼29%, 38 of 133). Indeed, protracted loss of H3K27ac at promoters was linked to a severe defect in transcriptional silencing in mutant cells (Figures 4C and 4D). Together, our data show that HDAC3 mediates efficient transcriptional silencing of the majority of genes through deacetylating histones. However, it remains unclear how HDAC3 is targeted to the X chromosome to help silencing.

### HDAC3 Is Pre-bound on the X Chromosome, Likely to Promote Deacetylation during XCI

**Figure 5:**
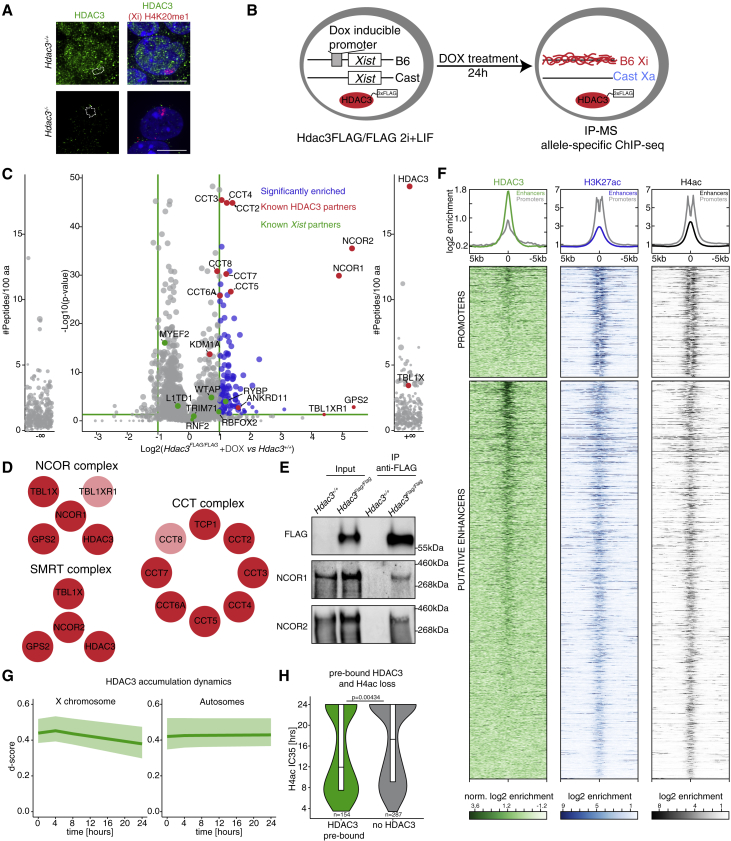
HDAC3 Is Pre-bound on the X Chromosome, Likely to Promote Efficient Histone Deacetylation (A) HDAC3 sub-nuclear localization in TX1072 (*Hdac3*^*+/+*^) and *Hdac3*^*−/−*^ ESCs induced with DOX for 24 hr. Xi was detected by H4K20me1 enrichment (dotted area) and shows no enrichment for HDAC3. Scale bar, 10 μm. (B) Schematic representation of the *Hdac3*^*FLAG/FLAG*^ ESC line, which expresses endogenous HDAC3 with a 3xFLAG tag. (C) Volcano plot analysis identifying interactors of HDAC3 in ESCs. Binding partners were obtained by using quantitative label-free mass spectrometry analysis performed from five replicates. Shown are the fold changes (*Hdac3*^*FLAG/FLAG*^ versus *Hdac3*^*+/+*^), quantified with an absolute fold change of 2 or more with an adjusted p value of ratio significance of 0.05 or less and with 3 or more peptides. Known HDAC3 (red) and *Xist* (green) interactors are shown. External plots show proteins with peptides identified only in one sample type (left in *Hdac3*^*+/+*^ and right in *Hdac3*^*FLAG/FLAG*^). (D) Representation of complexes identified as HDAC3 interactors, with pink proteins representing partners, which did not reach significance thresholds. (E) Immunoprecipitation; western blot validating HDAC3 interaction with both NCOR1 and NCOR2. (F) Heatmap of HDAC3 enrichment at all H3K27ac and H4ac peaks across the genome. Metaplots show average enrichment of HDAC3, H3K27ac, and H4ac at promoters and putative enhancers. For HDAC3, the signal was normalized to the no-FLAG ChIP-seq control. (G) HDAC3 accumulation dynamics (shading is the interquartile range) on peaks at the X chromosome (left) and autosomes (right). (H) Comparison of peak deacetylation (H4ac) dynamics (IC35) and HDAC3 pre-binding. The p value is from a Wilcoxon rank-sum test. See also Figure S5 and Table S2.

**Figure S5:**
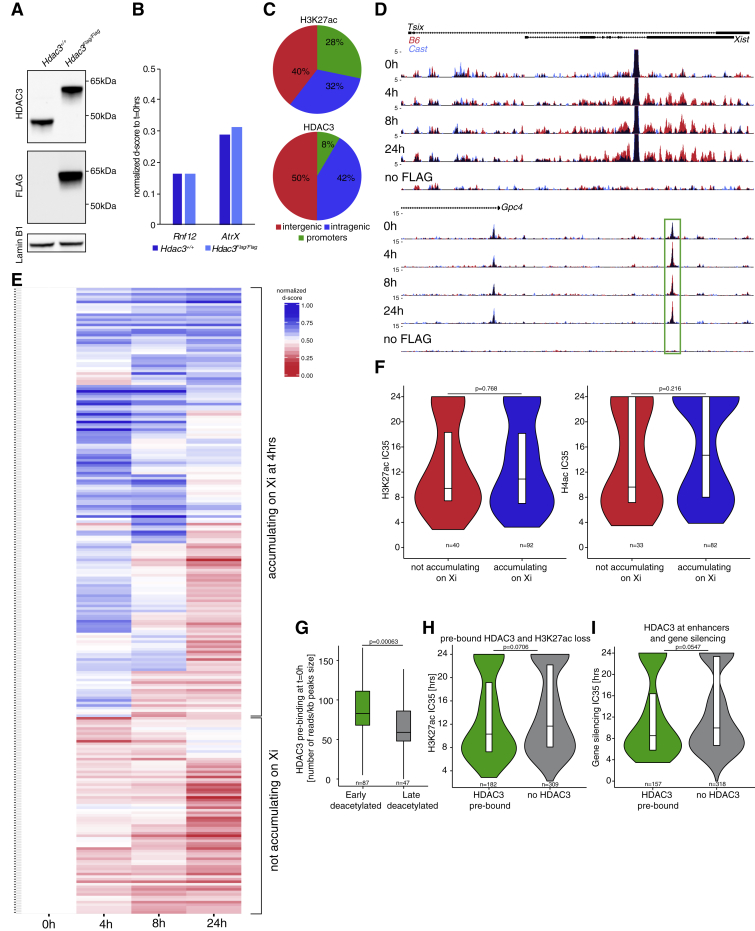
HDAC3 Is Pre-bound on the X Chromosome and Only Weakly Accumulates during XCI, Related to Figure 5 (A) Western blot analysis of *Hdac3*^*Flag/Flag*^ line in comparison to *Hdac3*^*+/+*^ ESC line probed with antibodies specific for FLAG, HDAC3 and Lamin B1. (B) Pyrosequencing validation of XCI efficiency in *Hdac3*^*Flag/Flag*^ compared to *Hdac3*^*+/+*^ ESC. Samples were collected after 0 and 24hrs of DOX treatment. (C) General annotation of all H3K27ac and HDAC3 peaks. (D) Genome browser track showing HDAC3 binding around *Xist* and *Gpc4*. Shown are allele-specific tracks (Cast-Xa: blue; B6-Xi: red). Green box points to HDAC3 peak becoming enriched for B6-Xi reads. Low-level accumulation of HDAC3 at the *Xist* locus is observed as well as skewing of 69% of HDAC3 peaks along the X chromosome. (E) Heatmap of normalized d-score for all HDAC3 peaks on the X chromosome. Peaks were clustered according to Xi (B6) read accumulation at 4hrs. (F) Violin plots showing the timing of deacetylation (left: H3K27ac; right: H4ac) at peaks accumulating HDAC3 on the Xi (B6) at 4hrs or not. (G) Boxplot showing the level of HDAC3 pre-bound at t = 0hr on the X chromosome at peaks deacetylated early and late. Outliers are not shown. (H) Violin plot showing timing of efficient H3K27ac loss (IC35) at regions pre-bound or not by HDAC3. (I) Violin plot showing timing of efficient transcriptional silencing (IC35) in relation to enhancer pre-binding by HDAC3. Promoter-enhancer pairs were identified as in Figure S1M. All p values were calculated using Wilcoxon rank sum test.

It has been proposed that HDAC3 might be either *de novo* recruited to the Xi by *Xist* through interaction with the SPEN protein (McHugh et al., 2015) or, alternatively, that *Xist-*coating might lead to the activation of already pre-bound HDAC3. To test these two hypotheses, we measured sub-nuclear localization of HDAC3 after 24 hr of DOX treatment. IF analysis revealed no HDAC3 enrichment on the Xi and even a slight depletion (Figure 5A). This surprising result prompted us to further explore the question of HDAC3 recruitment. To circumvent problems with antibody quality, we knocked in a 3xFLAG tag into the 3′ region of the endogenous *Hdac3* gene (Figure 5B). This novel ESC line (*Hdac3*^*FLAG/FLAG*^) expressed nearly normal levels of HDAC3 (Figure S5A) and robustly silenced the X chromosome, indicating that HDAC3 remained functional (Figure S5B).

We then set out to identify potential common interactors of *Xist* and HDAC3 in DOX-treated cells. To this end, we performed anti-FLAG immunoprecipitation followed by quantitative label-free mass spectrometry analysis (*Hdac3*^*FLAG/FLAG*^ versus *Hdac3*^*+/+*^ in five replicates) (Figure 5C). Among significant interactors, we identified nearly all components of the NCOR and SMRT complexes as well as their chaperone complex CCT (Figures 5D and 5E; Guenther et al., 2002). Intriguingly, of all *Xist* binding partners (Chu et al., 2015), only RYBP (a PRC1 factor) showed a weak binding to HDAC3 (Figure 5C). Our results confirm the previously reported interactions between HDAC3 and NCOR and SMRT but argue against strong HDAC3 recruitment by *Xist* RNA binding partners like SPEN.

To directly measure HDAC3 recruitment to the Xi, we performed anti-FLAG ChIP-seq prior to and during XCI. We found robust binding of HDAC3 to multiple H3K27ac and H4ac peaks throughout the genome (∼65% of HDAC3 peaks are enriched for H3K27ac [∼65% on the X chromosome] and ∼61% for H4ac [∼58% on the X chromosome]) (Figure 5F). The vast majority of HDAC3 peaks were intriguingly found to reside within putative enhancers rather than at gene promoters (Figure 5F; Figure S5C). Allele-specific analysis revealed a transient and modest enrichment after 4 hr of induction on the Xi (B6 reads) at the *Xist* locus and at some HDAC3 peaks (Figure 5G; Figures S5D and S5E). Together, this indicates that HDAC3 indirectly and unstably interacts with some of the *Xist* binding partners to allow for low-level and transient recruitment to the X chromosome. However, no significant correlation between HDAC3 *de novo* recruitment and timing of deacetylation was found (Figure S5F). On the other hand, the histone acetylation peaks that are lost early during XCI did show higher levels of HDAC3 pre-binding (Figure S5G). In fact, HDAC3-bound regions show more efficient (low IC35) deacetylation of H4ac and a similar but statistically not significant trend for H3K27ac (Figure 5H; Figure S5H). Finally, we observed a mild but statistically insignificant trend for HDAC3 pre-bound enhancers to surround rapidly silenced genes (Figure S5I). In summary, we show that HDAC3 does not appear to be robustly recruited *de novo* to the X chromosome via *Xist* RNA coating and does not strongly bind known partners of *Xist*. Rather, the majority of HDAC3 is pre-bound at putative enhancers, and, upon *Xist* induction, this results in efficient histone deacetylation, promoting transcriptional silencing.

### PRC1-Dependent H2AK119Ub Accumulation Precedes H3K27me3 Deposition

**Figure S6:**
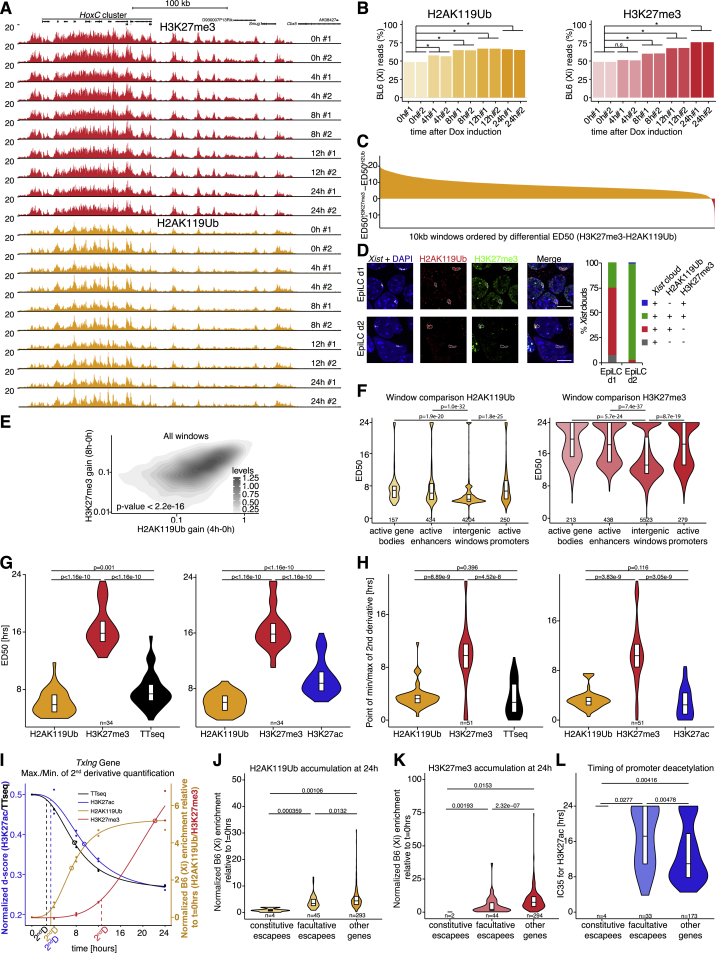
Allele-Specific Natic ChIP-Seq Monitors Changes in Representative Histone Modifications during XCI, Related to Figure 6 (A) Genome browser plots of H3K27me3 (red) and H2AK119Ub (yellow) enrichment at the *Hoxc* cluster. Shown are all mapping reads at all time points and biological duplicates. Note highly reproducible and stable pattern of enrichment. (B) Bar plot showing the percentage of B6 reads mapping to the X chromosome in the nChIP-seq time course. ^∗^p value < 0.05 calculated using Student’s t test. (C) Plot showing the distribution of differential ED_50_ (H3K27me3-H2K119Ub) for all 10kb windows along the X chromosome. Each bar represents a single window, these were ordered accordingly to their differential ED_50_. (D) IF/RNA FISH for *Xist* (gray), H2AK119Ub (red) and H3K27me3 (green) in day 1 or 2 EpiLCs. White dotted lines outline *Xist* domains. Bar plot quantifies PcG-mark enrichment at 100 *Xist* clouds. Scale bar = 10 μm. (E) Density plots correlating H2AK119Ub gain (4 versus 0hrs) with H3K27me3 gain (8 versus 4hrs). Shown is the behavior of all 10kb windows spanning the X chromosome. All scales are logarithmic. p value was calculated using Pearson correlation test. The correlation coefficient is ρ = 0.622 (F) Violin plots showing the distribution of ED_50_ for H2AK119Ub (left) and H3K27me3 (right) at windows mapping to the active gene bodies, active enhancers, intergenic regions and active promoters. p values calculated using Wilcoxon rank sum test. (G) ED_50_ analysis of H2AK119Ub (yellow), H3K27me3 (red) accumulation at gene bodies in comparison to gene silencing (black, left) and promoter H3K27ac loss (blue, right). p values calculated using paired Wilcoxon rank sum test. (H) Analysis of the point when sigmoid’s second derivative reaches max/min. This is a proxy for when H2AK119Ub (yellow) and H3K27me3 (red) start to accumulate at gene bodies in comparison to the beginning of gene silencing (black, left) and promoter H3K27ac loss (blue, right). p values calculated using paired Wilcoxon rank sum test. (I) Dynamics of H2AK119Ub (yellow), H3K27me3 (red) accumulation, promoter deacetylation (H3K27ac, blue) and transcriptional silencing (black) of an X-linked gene: *Txlng*. Empty dots present ED_50_ of sigmoid fittings, while perpendicular lines max/min of second derivative (2ndD). Left axis of normalized d-score is for H3K27ac and TT-seq dynamics, right axis of normalized B6-read accumulation is for H2AK119Ub and H3K27me3. (J-K) Violin plots showing H2AK119Ub (J) and H3K27me3 (K) accumulation at gene bodies of constitutive and facultative escapees when compared to remaining genes. Shown is the normalized enrichment of B6-reads after 24hrs of DOX treatment when normalized to t = 0hr. p values calculated using Wilcoxon rank sum test. (L) Violin plot showing H3K27 deacetylation dynamics (IC35) at promoters of constitutive and facultative escapees when compared to remaining genes. p values calculated using Wilcoxon rank sum test.

**Figure 6:**
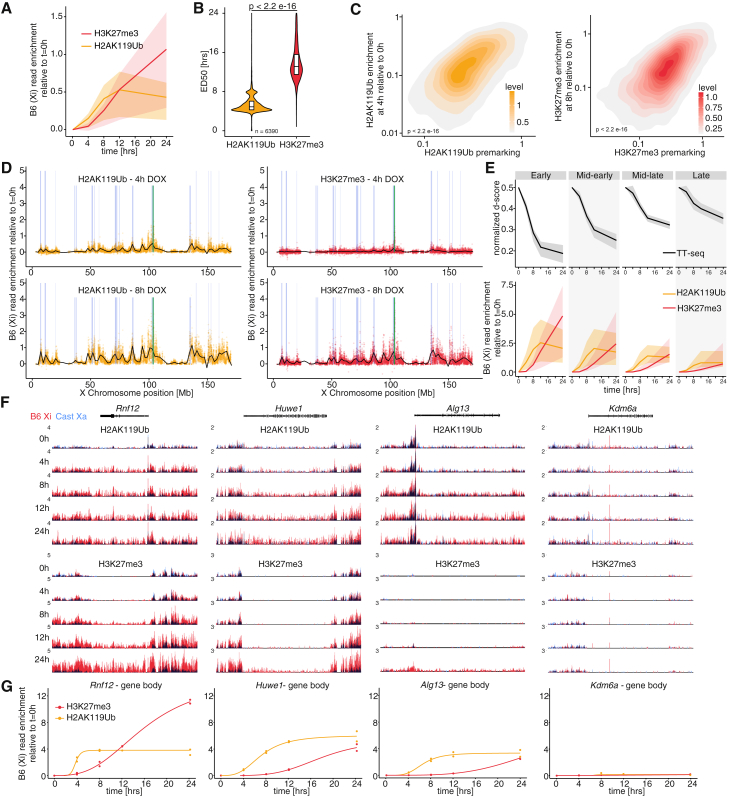
PRC1-Dependent H2AK119Ub Accumulation Precedes H3K27me3 Deposition (A) Quantification of average H2AK119Ub (yellow) and H3K27me3 (red) enrichment at the Xi (B6 allele) compared with t = 0 hr in 10-kb windows spanning the whole chromosome. Shading is the interquartile range. (B) Pairwise comparison of H2AK119Ub and H3K27me3 accumulation dynamics (ED_50_) at the X chromosome. All 10-kb windows with ED_50_ < 24 hr are plotted. The p value is from a paired Wilcoxon rank sum test. (C) Correlation between H2AK119Ub (left) or H3K27me3 (right) pre-marking (t = 0 hr) and *de novo* enrichment of those marks shortly after *Xist* RNA induction (t = 4 hr for H2AK119Ub and t = 8 hr for H3K27me3). Plotted are all 10-kb windows spanning the X chromosome. All scales are logarithmic. The p values are from Pearson’s correlation test with ρ = 0.538 (H2AK119Ub) and 0.441 (H3K27me3). (D) H2AK119Ub (yellow) and H3K27me3 (red) accumulation dynamics across the Xi after 4 and 8 hr of DOX treatment. The black line is a locally estimated scatterplot smoothing (LOESS) regression on all 10-kb windows (dots). Shown is the *Xist* locus (green) and *Xist* entry sites (blue). (E) Dynamics of transcriptional silencing (top) and H2AK119Ub (yellow) and H3K27me3 (red) accumulation at gene bodies. Genes were separated based on transcriptional silencing dynamics. Shading is the interquartile range. (F) Genome browser tracks showing H2AK119Ub (top) and H3K27me3 (right) accumulation at genes silenced rapidly (*Rnf12*), slowly (*Huwe1, Alg13*), or not at all (*Kdm6a*). Allele-specific tracks were overlaid (B6 in red and Cast in blue). (G) Quantification of PcG mark accumulation at gene bodies (from F) with sigmoidal curves fitted. See also Figure S6.

Repressive chromatin modifications are also known to appear shortly after *Xist* RNA coating, in particular H2AK119Ub (PRC1-dependent) and H3K27me3 (PRC2-dependent). However, the early dynamics of this process, as well as the relative timing of H3K27me3 and H2AK119Ub deposition, have remained largely unexplored. To address this question, we have performed nChIP-seq for both PcG-dependent marks. These datasets showed a reproducible and stable pattern of enrichment on autosomes, both between time points and biological replicates (Figure S6A). In contrast, the X chromosome showed a bias in H2AK119Ub enrichment toward the B6 allele (Xi) after 4 hr of DOX induction and after 8 hr in the case of H3K27me3 (Figure S6B). First, we analyzed relative B6-read enrichment within 10-kb windows across the whole X chromosome normalized to t = 0 hr and observed rapid deposition of H2AK119Ub preceding that of H3K27me3 (Figure 6A). To quantify the dynamics at individual windows, we extracted the ED_50_ parameter, as was done for active marks, but using B6-read accumulation (STAR Methods; Figure S1F). Pairwise comparison of ED_50_ at all 10-kb windows across the X chromosome showed that H2AK119Ub accumulates most efficiently prior to H3K27me3 (Figure 6B), with very few exceptions (∼1%) (Figure S6C).We also validated this finding using IF/RNA FISH during ESC differentiation toward EpiLCs (Figure S6D). Evidently, the Xi becomes enriched for H2AK119Ub prior to the PRC2-dependent H3K27me3 across the whole chromosome, although there may be local differences in the dynamics of this process. Indeed, certain regions preferentially accumulate Polycomb marks already at early time points (i.e., by 4 hr for H2AK119Ub or 8 hr for H3K27me3; Figure 6D). This early accumulation was found to be prominent around the *Xist* locus (green line) as well as the first regions with which it associates (“entry sites,” blue lines) (Engreitz et al., 2013). There is also significant overlap between H2AK119Ub and H3K27me3 patterns of accumulation, and, interestingly, these primary deposition sites are typically marked by PcG prior to *Xist* upregulation (Figure 6C, Figure S6E). Thus, most H2AK119Ub accumulation occurs at sites pre-marked by PcG and rapidly coated by *Xist*. Subsequent H3K27me3 deposition closely follows this early deposition pattern.

We next investigated the accumulation dynamics of both marks at specific types of genomic windows. ED_50_ analysis revealed that H2AK119Ub accumulates first intergenically and is deposited only later at active gene bodies, promoters, and putative enhancers (Figure S6F). This pattern of accumulation is tightly followed by H3K27me3 deposition and spread. What is more, PcG-dependent mark accumulation seems to be closely linked to gene silencing kinetics (Figure 6E). This is exemplified by tracks for genes that are either silenced rapidly (*Rnf12*) or slowly (*Huwe1* and *Alg13*) or escape the process altogether (*Kdm6a*) (Figures 6F and 6G). Direct comparison of gene silencing and promoter deacetylation dynamics with PcG mark accumulation at gene bodies revealed a significantly lower ED_50_ parameter for H2AK119Ub (Figure S6G). This indicates that the H2AK119Ub deposition reaches its maximum rate earlier than gene silencing and promoter deacetylation. Next, we measured the time when the sigmoid’s second derivative reaches its minimum or maximum. We use this parameter as a proxy for when the curve starts to increase or decrease (Figure S6I). This revealed that gene silencing, promoter deacetylation (H3K27ac loss), and H2AK119Ub accumulation at gene bodies are all initiated at the same time, whereas H3K27me3 is significantly delayed (Figure S6H). Thus, we see dynamic spreading of H2AK119Ub from intergenic regions to gene bodies coinciding with transcriptional silencing.

We also investigated genes that normally escape from XCI (constitutive) or escape more variably and in specific tissues (facultative escapees) (Disteche and Berletch, 2015). We examined a highly expressed constitutive escapee (*Kdm6a*; Figures 6F and 6G) and found no PcG-dependent mark accumulation at its promoter or gene body in our dataset. In line with this, both constitutive and facultative escapees show no or lower accumulation of H2AK119Ub and H3K27me3 at their gene bodies (Figures S6J and S6K). This is consistent with significantly delayed H3K27ac loss at their promoters (Figure S6L). Thus, genes escaping transcriptional silencing do not undergo promoter deacetylation and do not accumulate PcG-associated marks.

All in all, our analysis reveals the hierarchy of PcG marks during XCI, with H2AK119Ub accumulating significantly prior to H3K27me3. This process is first initiated at large chromatin domains that are pre-marked by PcG (megabase scale) and that correspond to some of the first regions with which *Xist* RNA interacts (Engreitz et al., 2013). We demonstrate that PcG marks accumulate first intergenically and subsequently spread into regulatory elements and gene bodies. This spread into genes coincides with transcriptional silencing, raising the question of whether the two processes are functionally linked.

### Transcriptional Silencing Is Necessary for PcG Spreading

**Figure 7:**
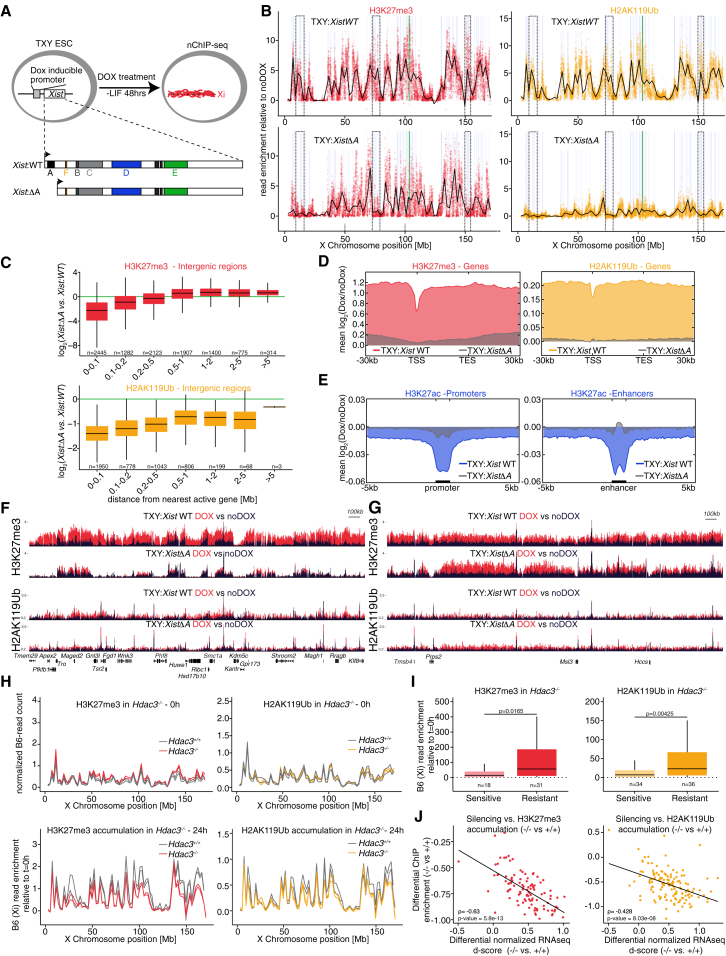
PcG Spreading along the X Chromosome Requires Transcriptional Silencing (A) Schematic representation of the experimental design. By DOX addition, male TXY mouse ESC lines allow expression of *Xist* or *Xist:ΔA*. (B) H3K27me3 (red) and H2AK119Ub (yellow) accumulation across the Xi after 48 hr of DOX compared with no DOX in WT (top) and *Xist:ΔA* (bottom) cells. Each dot represents a single 10-kb window. The black line indicates a LOESS regression on all windows. Shown are the *Xist* locus (green line), active genes (light blue), and examples of gene-dense regions (dotted boxes). (C) Correlation between the distance from an active gene and the H3K27me3 (top) or H2AK119Ub (bottom) accumulation in *Xist:ΔA* cells compared with WT cells. (D and E) Average plots showing accumulation or depletion of H3K27me3 (D, left), H2AK119Ub (D, right), and H3K27ac (E) over all X-linked active genes (D), promoters (E), or enhancers (E) in *Xist:ΔA*- and *Xist:WT*-expressing cells. (F and G) Genome browser plots showing H3K27me3 and H2AK119Ub enrichments in regions with a high (F) or low (G) density of active genes. Signals from DOX-treated (red) and untreated (blue) samples were overlaid. Plotted are only expressed genes. (H) H3K27me3 (left) and H2AK119Ub (right) enrichment across the Xi at 0 hr (top) and their accumulation at 24 hr (bottom) in *Hdac3*^*+/+*^(TX1072, gray) and *Hdac3*^*−/−*^ cells. Lines indicate LOESS regression of 10-kb windows spanning the whole X chromosome. (I) Quantification of H3K27me3 (red) and H2AK119Ub (yellow) accumulation on gene bodies at 24 hr compared to 0 hr in *Hdac3*^*−/−*^. Genes were separated based on their sensitivity to HDAC3 loss. The p values are from a Wilcoxon rank-sum test. (J) Correlation between the transcriptional silencing defect and H3K27me3 (red) and H2AK119Ub (yellow) accumulation defect in *Hdac3*^*−/−*^ compared with *Hdac3*^*+/+*^(TX1072). The black line represents linear regression fitting. ρ and p values from Pearson's correlation are shown. See also Figure S7.

To address whether spreading of these repressive histone modifications from intergenic regions into genes is a cause or consequence of transcriptional silencing, we decided to investigate PcG mark distribution in a *Xist* mutant lacking the A-repeat element (*Xist:ΔA*). *Xist:ΔA* RNA is able to coat the X chromosome and lead to PcG mark accumulation (based on IF) but cannot initiate gene silencing (Kohlmaier et al., 2004, Sakata et al., 2017, Schoeftner et al., 2006, Wutz et al., 2002). *Xist:ΔA* thus allows the relationship between repressive chromatin changes and transcriptional repression to be addressed. For this, we used previously published male ESCs harboring wild-type (TXY:*Xist*) or mutant *Xist* (TXY:*XistΔA*) under a DOX-inducible promoter at the endogenous locus (Wutz et al., 2002; Figure 7A). These ESCs do not require allele-specific analysis, as all X-mapping reads will originate from the single X chromosome. After collecting DOX-treated and untreated differentiated cells (48 hr), we performed nChIP-seq for three histone modifications (H3K27ac, H3K27me3, and H2AK119Ub) in biological duplicates. Samples were normalized for ChIP quality prior to further analysis (STAR Methods).

**Figure S7:**
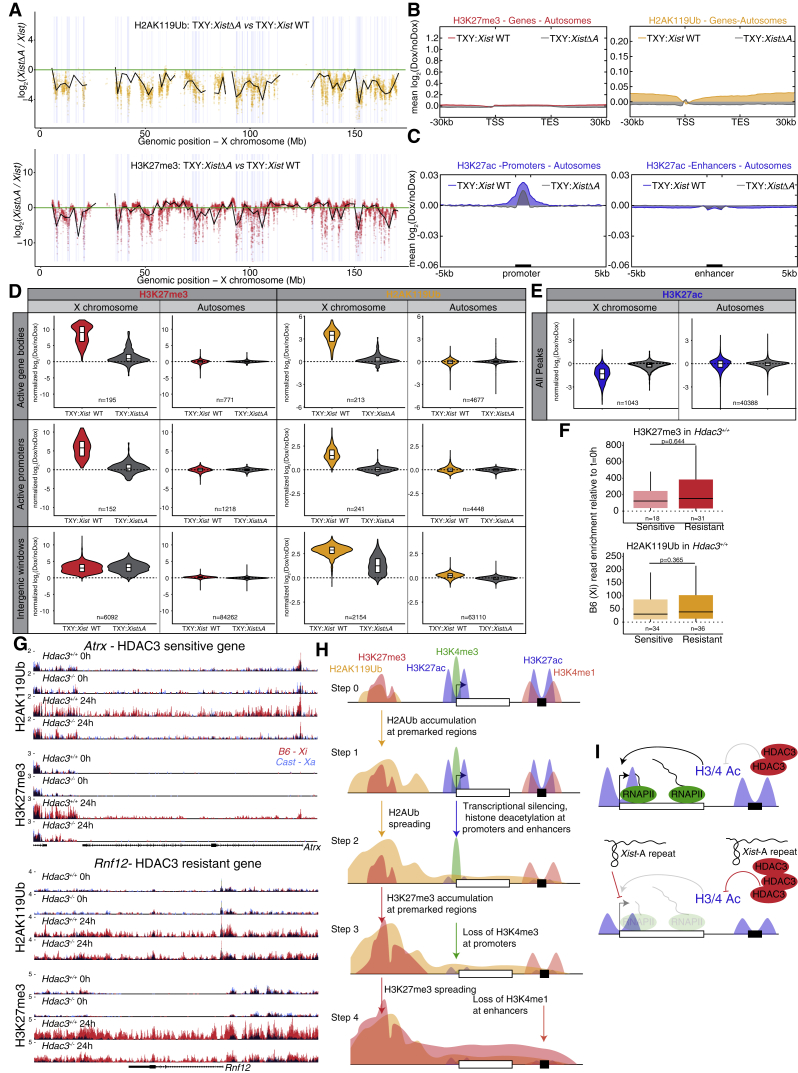
Dependence of PcG Spreading on Gene Silencing; Proposed Models, Related to Figure 7 (A) Plots showing H3K27me3 (red) and H2AK119Ub (yellow) differential accumulation between *Xist:ΔA* and *Xist:WT* samples. Shown is average log2 fold change enrichment. Each dot represents a single 10kb window. Black line is a loess regression on all windows, active genes are in light blue. (B) Average plots showing average accumulation of H3K27me3 (left) and H2AK119Ub (right) over autosomal active genes ± 30kb in *Xist:ΔA* cells compared to *Xist:WT.* Shown is the mean normalized log2 enrichment of doxycycline versus no-doxycycline samples. (C) Average H3K27ac plots over autosomal promoters and enhancers (+/−5kb) in *Xist:ΔA* and *Xist:WT* expressing cells. Shown is the normalized mean log2 enrichment of doxycycline versus no-doxycycline samples. (D) Violin plots quantifying H3K27me3 and H2AK119Ub accumulation over active gene bodies, promoters and intergenic regions. All windows were split into X-linked and autosomal. (E) Violin plots quantifying H3K27ac depletion at its peaks. All peaks were split into X-linked and autosomal. (F) Boxplots quantifying H3K27me3 (red) and H2AK119Ub (yellow) accumulation on gene bodies at 24hrs compared to 0 hr in *Hdac3*^*+/+*^. Shown are only B6 (Xi) accumulated reads. Genes were separated based on their sensitivity to HDAC3 loss. P values were calculated using Wilcoxon rank sum test. (G) Genome browser tracks showing H2AK119Ub and H3K27me3 accumulation at a gene sensitive (*AtrX;* top) or resistant (*Rnf12;* bottom) to HDAC3 loss. B6- and Cast-specific reads are shown in red and blue. Allele-specific tracks were overlaid. (H) Stepwise model of the epigenomic roadmap for XCI. Step 1: H2AK119Ub accumulates intergenically at sites of *Xist* binding, which are pre-marked by PcG. This stage is complete by 4 hr. Step 2: Concurrently with the initiation of transcriptional silencing, H3K27 starts to be deacetylated at promoters and H2AK119Ub commences to spread into gene bodies. Both transcriptional silencing and histone deacetylation are entirely dependent on the A-repeat of *Xist*. Moreover, HDAC3 is a key player involved in promoting efficient transcriptional silencing by deacetylating promoters and enhancers. This step is complete for early silenced genes by 4 hr, while other genes will typically reach this point by 8hrs. H2AK11Ub spreading into genes necessitates the initiation of gene silencing. Step 3: Decommissioning of promoters by H3K4me3 loss follows a protracted dynamic. H3K27me3 deposition at intergenic regions is also delayed when compared to H2AK119Ub enrichment. These events take place around the 8hrs time point. Step 4: Even more delayed is the decommissioning of enhancers by the loss of H3K4me1, indicating that it is the consequence of gene silencing rather than its cause. Similarly, H3K27me3 spreading into genes occurs after, and is dependent on, gene silencing. This indicates that its role in initiating transcriptional silencing is unlikely but does not preclude its importance in XCI maintenance. Rapidly silenced genes can reach this point already after 8 hr of DOX treatment, while other genes need 24 hr. (I) Model of the involvement of HDAC3 in XCI. Gene silencing is mediated mainly through the HDAC3, which is pre-bound at enhancers already prior to *Xist* upregulation. Upon XCI induction, HDAC3 is only modestly recruited to the Xi, while the pre-bound HDAC3 presumably becomes activated. Full activation of HDAC3 is entirely dependent on the *Xist* A-repeat region as no deacetylation is observed along the Xi when this element is deleted. How *Xist* A-repeat achieves this remains unclear. Most likely HDAC3 activation occurs thanks to *Xist* A-repeat interaction with SPEN, and in turn, this has been proposed to recruit SMRT (Chu et al., 2015, McHugh et al., 2015, Monfort et al., 2015). We observe that HDAC3 in the context of mouse ESC is strongly bound to both SMRT and NCOR components. Such SPEN-SMRT interaction could allow for HDAC3 mediated histone deacetylation and efficient gene silencing (McHugh et al., 2015).

First, we investigated the relationship between PcG-associated mark spread and transcriptional silencing in WT and *Xist:ΔA* cells. We observed that most regions of the X chromosome recruit nearly normal levels of H3K27me3 in the *Xist:ΔA* cells. However, clusters of active genes are a notable exception to this (Figure 7B; Figure S7A). The same regions are also found not to accumulate H2AK119Ub. Intriguingly, reduced recruitment of H2AK119Ub was observed throughout the chromosome in *Xist:ΔA* cells compared with WT *Xist* (Figure 7B). We next quantitated the deposition of both Polycomb marks in relation to regions with actively transcribed genes (Figure 7C). Indeed, both marks show negligible accumulation in the *Xist:ΔA* mutant when within 100 kb from active genes. On the other hand, regions more distal (a minimum of 200 kb away) to transcribed loci gain normal levels of H3K27me3 and intermediate levels of H2AK119Ub, as exemplified in genome browser tracks (Figures 7F and 7G). Thus, *Xist:ΔA* leads to PRC2 and PRC1 recruitment to regions without active transcription; however, both complexes seem impeded from spreading into domains of active genes. In line with this, we observed no H3K27me3 or H2AK119Ub enrichment within promoters and bodies of active genes (Figure 7D; Figures S7B–S7E).

Our finding that H3K27 deacetylation promotes gene silencing during XCI prompted us to investigate H3K27ac loss at gene regulatory elements upon coating by *Xist:ΔA* RNA (Figure 7E; Figures S7C and S7E). Importantly, we did not observe significant deacetylation of either promoters or enhancers in the *Xist:ΔA* mutant. This was in stark contrast to robust inactivation of such regulatory elements when using full-length *Xist*. Thus, promoter and enhancer deacetylation are tightly linked to transcriptional silencing and dependent on the *Xist* A-repeat. This is in line with our HDAC3 experiments indicating a functional role for histone deacetylation in initiating gene repression.

To further assess how PcG accumulation relates to histone deacetylation and gene silencing, we performed nChIP-seq in *Hdac3*^*−/−*^ cells. We mapped H2AK119Ub and H3K27me3 in two independent *Hdac3*^*−/−*^ clones at 0 and 24 hr of DOX treatment. Although HDAC3 loss results in inefficient transcriptional silencing, it should not have major direct effects on PcG marks. Indeed, we found that the pattern of both PcG marks at t = 0 hr was very similar along the X chromosome (Figure 7H). After 24 hr of DOX treatment, both marks accumulate globally along the X chromosome in a similar pattern to that observed in *Hdac3*^*+/+*^ cells (Figure 7H), albeit with slightly lower efficiency. This result differs from the patterns observed in *Xist* A-repeat mutant cells because, in *Hdac3*^*−/−*^ cells, even gene-rich regions accumulate both PcG-associated marks. We then focused on what happens at genic regions. This revealed that genes that were not silenced in *Hdac3*^*−/−*^ cells (i.e., sensitive) accumulate neither H2AK119Ub nor H3K27me3 (Figure 7I; Figure S7F). On the other hand, genes efficiently repressed in *Hdac3*^*−/−*^ (resistant) robustly accumulated both marks. These relationships are exemplified by a *AtrX* (resistant gene) and *Rnf12* (sensitive gene) (Figure S7G). Thus, *Xist* coating and intergenic PcG mark deposition are not sufficient for H2AK119Ub spread into active genes. Only the induction of transcriptional silencing enables H2AK119Ub spread to occur.

In conclusion, we uncoupled the general deposition of the Polycomb marks over the X chromosome from transcriptional silencing. By doing so, we demonstrate that neither PRC1 nor PRC2 dependent marks are able to spread into actively transcribed regions. In the A-repeat mutant, this may be a result of defective *Xist* spreading or the presence of transcription (Engreitz et al., 2013). On the other hand, our experiments in *Hdac3*^*−/−*^ cells revealed that reduced transcription and histone deacetylation are required to enable PcG spreading into actively transcribed regions.

## Discussion

X chromosome inactivation is a powerful model for the study of transcriptional repression and the formation of facultative heterochromatin. The link between chromatin changes and transcriptional silencing during XCI has remained rather elusive. In this study, we provide the detailed choreography of early transcriptional and chromatin changes during the initiation of XCI at high molecular and temporal resolution (Figure S7H). We also explore the chromatin regulatory pathways that help to achieve efficient gene repression. All in all, our findings point to multiple parallel epigenetic mechanisms being at play during the initiation of XCI to ensure rapid and robust transcriptional silencing.

### *Xist* RNA as the Architect for Early Chromatin Changes along the X Chromosome

Our results indicate that the dynamics and efficiency of chromatin alterations along the X chromosome are, in part, shaped by the spreading of *Xist* RNA itself. Indeed, rapid histone deacetylation is most efficient in regions proximal to the *Xist* locus, in line with previous allele-specific RNA-seq experiments (Borensztein et al., 2017, Marks et al., 2015).The PcG-dependent marks accumulate first at regions pre-marked by H3K27me3 and H2AK119Ub, which were previously identified as *Xist* entry sites (XESs), lying in 3D proximity to the *Xist* locus (Engreitz et al., 2013). This striking PcG mark enrichment and 3D proximity are reminiscent of the Polycomb-enriched regions specifically interacting together in *Drosophila melanogaste*r (Cheutin and Cavalli, 2014). Thus, it is an exciting possibility that the chromatin landscape of the X chromosome prior to its inactivation instructs the folding of the chromosome, resulting in a specific pattern of *Xist* spreading and subsequent dynamics in gene silencing.

### Spreading of PcG Marks, but Not Their Initial Recruitment, Requires Gene Silencing

Our high-resolution XCI analysis also sheds light on the kinetics of PcG mark deposition and spreading along the X chromosome. The very rapid accumulation of PRC1-dependent H2AK119Ub that occurs prior to the deposition of H3K27me3 is in contrast with some previous reports suggesting direct PRC2 recruitment by the *Xis*t RNA (Zhao et al., 2008) but is in line with recent reports where a non-canonical PRC1 complex (including PCGF3 and PCGF5) is first recruited, and this then recruits PRC2 (Almeida et al., 2017). Using *Xist* transgenes on autosomes, this was proposed to occur through HNRNPK bridging the B-repeat region of *Xis*t with PRC1 (Pintacuda et al., 2017). This finding appears to be contradicted by the defect in H2AK119Ub deposition in the *Xist* A-repeat mutant. However, we propose that transcriptional silencing may be required to allow for secondary canonical PRC1 recruitment. Indeed, initial H2AK119Ub enrichment is thought to recruit the PRC2 complex via JARID2 or AEBP2, allowing H3K27me3 accumulation, which, in turn, would allow canonical PRC1 binding (da Rocha et al., 2014). Further studies of how transcription affects this feedback and of the timing of PRC1 and PRC2 recruitment are necessary to further validate the revised model.

Here we find that, during the first hours of XCI, PcG marks initially accumulate at intergenic regions and later spread into genes. In cells expressing the *Xist* A-repeat mutant, neither PRC1 nor PRC2-dependent marks are able to spread into genic regions because they are probably impeded by ongoing transcription (Kaneko et al., 2014). Alternatively, the *Xist* A-repeat deletion might lead to local defects in *Xist* spreading, as suggested by Engreitz et al. (2013). Furthermore, our detailed analysis of *Hdac3* mutant cells revealed that H2AK119Ub spreading to genes requires their—at least partial—deacetylation and transcriptional inactivation. This indicates that PRC1 mark spreading is not the trigger for initiating gene repression. On the other hand, because both processes seem to occur around the same time, there might be a potential role of PRC1 in facilitating efficient XCI but not initiating it. Indeed, in an autosomal context, a mutant of *Xist*, which is unable to recruit PRC1, has slightly reduced silencing capacity (Almeida et al., 2017, Pintacuda et al., 2017).

### Histone Deacetylation Has an Early Role in Gene Silencing Events during XCI

Here we identify H3K27 deacetylation as one of the earliest chromatin alterations during XCI, tightly linked to transcriptional silencing. This is in line with previous IF/FISH studies in differentiating ESCs and embryos (Chaumeil et al., 2002, Okamoto et al., 2004). Moreover, by using *Hdac3* loss-of-function experiments, we demonstrate that histone deacetylation is not simply due to reduced transcription. Rather, it appears to be an active process that promotes gene silencing. We propose a model whereby histone deacetylation by the pre-bound HDAC3 promotes efficient silencing of most X-linked genes (Figure S7I). A previous study has shown that *Hdac3* knockdown results in defective silencing of a single locus during XCI (McHugh et al., 2015). However, our study reveals that other *Xist* A-repeat-dependent but HDAC3-independent mechanisms are also at play during XCI. This is presumably to safeguard and mediate timely gene repression, especially at genes that need to become rapidly silenced; e.g., *Rnf12*. Although we have excluded the involvement of other histone deacetylases during XCI initiation, future studies will reveal whether these HDAC3-independent mechanisms act via other chromatin-modifying or -remodeling activities or whether they occur through entirely different pathways.

## STAR★Methods

### Key Resources Table

**Table:** 

REAGENT or RESOURCE	SOURCE	IDENTIFIER
**Antibodies**		

anti-LaminB1	Abcam	Cat#ab16048
anti-HDAC3	Santa Cruz	Cat#sc-376957 X
Anti-H3K4me3	Merck-Millipore	Cat#05-1339
anti-H3K4me1	Cell Signaling	Cat#5326S
anti-H3K27ac	Active Motif	Cat#39685
anti-H3K27me3	Cell Signaling	Cat#9733S
anti-H3K9ac	MBL Lifescience	Cat#MABI0305
anti-pentaH4ac	Merck-Millipore	Cat#06-946
anti-H2AK119Ub1	Cell Signaling	Cat#8240S
anti-NCOR1	Abcam	Cat#ab3482
anti-NCOR2	Abcam	Cat#ab5802
anti-FLAG	Sigma-Aldrich	Cat#F1804-50UG

**Chemicals, Peptides, and Recombinant Proteins**		

RGFP966	Abcam	Cat#ab144819
BRD6688	Bertin Pharma	Cat#19836.1 mg
EZ-Link HPDP-Biotin	ThermoFisher Scientific	Cat#21341
4-Thiouridine	Carbosynth	Cat#NT06186
Tasquinimod	Bertin Pharma	Cat#17692
Tubastatin A	Bertin Pharma	Cat#10559
PCI-34051	Bertin Pharma	Cat#T6325
LMK235	Bertin Pharma	Cat#14969
Trypsin/LysC Mix, Mass Spec Grad	Promega	Cat#v5071

**Critical Commercial Assays**		

PyroMark Gold Q24	QIAGEN	Cat#970802
Ovation Ultralow System V2 1-16	Nugen Technologies	Cat#0344-32
Ovation Universal RNA-Seq System	Nugen Technologies	Cat#7102-08
Ovation Mouse RNA-Seq System 1-16	Nugen Technologies	Cat#348
Nick Translation Kit	Roche	Cat#10976776001
MODified Histone Peptide Array	Active Motif	Cat#13005
TruSeq Stranded Total RNA Library Prep Kit with Ribo-Zero Human/Mouse/Rat	Illumina	Cat# RS-122-2201
μMACS Streptavidin Kit	Miltenyi Biotec	Cat# 130-074-101
miRNeasy Micro kit	QIAGEN	Cat# 217084
MEGAscript T7 Transcription Kit	ThermoFisher Scientific	Cat# AM1333

**Deposited Data**		

TT-seq; RNA-seq; ChIP-seq	This study	GSE116480
IP-MS	This study	PXD011344

**Experimental Models: Cell Lines**		

mouse: ESC TX1072	Heard Lab	(Schulz et al., 2014)
mouse: ESC TX1072-Hdac3−/−#1	Heard Lab	this study
mouse: ESC TX1072-Hdac3−/−#2	Heard Lab	this study
mouse: ESC TX1072-Hdac3Flag/Flag	Heard Lab	this study
mouse: ESC TXY:XistWT	Wutz Lab	(Wutz et al., 2002)
mouse: ESC TXY:XistΔA	Wutz Lab	(Wutz et al., 2002)

**Oligonucleotides**		

Primers	This study	See Table S3

**Recombinant DNA**		

pSpCas9n(BB)-2A-Puro (PX462)	Feng Zang Lab	Addgene #Cat:48141
p510	Avner Lab	(Chaumeil et al., 2002)
AtrX Fosmid probe	BacPac Consortium at Children’s Hospital Oakland Research Institute	Cat#WI1-2039P10
Huwe1 Fosmid probe	BacPac Consortium at Children’s Hospital Oakland Research Institute	Cat#RP24-157H12
TT-seq Spike-ins	Cramer Lab	See Table S4

**Software and Algorithms**		

Bowtie2 (2.2.5)	(Langmead and Salzberg, 2012)	http://bowtie-bio.sourceforge.net/bowtie2/index.shtml
TrimGalore (v0.4.0)	Felix Krueger lab	https://github.com/FelixKrueger/TrimGalore
Cutadapt (1.8.2)	(Martin, 2011)	https://github.com/marcelm/cutadapt
SNPSplit (0.3.2)	(Krueger and Andrews, 2016)	http://www.bioinformatics.babraham.ac.uk/projects/SNPsplit/
Bedtools (2.25.0)	(Quinlan and Hall, 2010)	http://bedtools.readthedocs.io/en/latest
MACS2 (2.0.10)	(Zhang et al., 2008)	https://github.com/taoliu/MACS/
Subread - featureCounts (1.5.1)	(Liao et al., 2014)	http://subread.sourceforge.net/
DeepTools (3.0.2)	(Ramírez et al., 2014)	http://deeptools.ie-freiburg.mpg.de
STAR (2.3.0)	(Dobin and Gingeras, 2015)	https://github.com/alexdobin/STAR
Samtools (1.3)	(Li et al., 2009)	https://github.com/samtools/samtools
Tophat (2.1.0)	(Trapnell et al., 2009)	https://github.com/infphilo/tophat
R (3.4.0)	R Core Team	http://www.R-project.org/
edgeR (R package)	(Robinson et al., 2010)	https://bioconductor.org/packages/release/bioc/html/edgeR.html
Limma (R package)	(Ritchie et al., 2015)	http://bioconductor.org/packages/release/bioc/html/limma.html
drc (R package)	(Ritz et al., 2015)	https://cran.r-project.org/web/packages/drc/index.html
Proteome Discoverer (v 2.2)	ThermoFisher Scientific	N/A
myProMS	(Poullet et al., 2007)	N/A
Bowtie2 (2.2.5)	(Langmead and Salzberg, 2012)	http://bowtie-bio.sourceforge.net/bowtie2/index.shtml
TrimGalore (v0.4.0)	Felix Krueger lab	https://github.com/FelixKrueger/TrimGalore

**Other**		

Xist oligo probe	Roche	Custom made
S220 Focused-ultrasonicator	Covaris	S220
HiSeq	Illumina	2500
Column LC Acclaim PepMap 100 C18	ThermoFisher Scientific	Cat# 164942 and 164535
Mass Spectrometer	ThermoFisher Scientific	Orbitrap FusionTribrid
Liquid Chromatography System	ThermoFisher Scientific	UltiMate 3000 RSLCnano

### Contact for Reagent and Resource Sharing

Further information and requests for resources and reagents should be directed to and will be fulfilled by the Lead Contact, Edith Heard (edith.heard@curie.fr)

### Experimental Model and Subject Details

#### Cell Lines

TX1072 (mouse, female, [*Mus musculus castaneus X* C57BL/6] embryonic stem cells) cells have been previously derived in the lab (Schulz et al., 2014). *Hdac3*^*−/−*^ and *Hdac3*^*Flag/Flag*^ clones have been derived from the TX1072 line by CRISPR/Cas9 targeting. Cells were cultured on gelatine-coated plates in Dulbecco’s Modified Eagle Medium (DMEM) supplemented with 15% fetal bovine serum, 2-mercaptoethanol (0.1mM), LIF(1000u/mL) and 2i (PD0325901 [0.4 mM], CHIR99021 [3 mM]) . TXY:*Xist* and TXY:*XistΔA* (mouse, male embryonic stem cells) were obtained from the Wutz team (Wutz et al., 2002). These lines were cultured on gelatine-coated plates in Dulbecco’s Modified Eagle Medium (DMEM) supplemented with 15% fetal bovine serum, 2-mercaptoethanol (50mM) and LIF (1000u/mL). All cells were incubated at 37°C with 8% CO_2_.

### Method Details

#### Doxycycline and inhibitor treatment

TX1072 based ESC lines were plated at a density of 1.8mln/T75 flask. After 24hrs of culture in DMEM/15%FCS+LIF+2i cell the medium was supplemented with doxycycline (1ug/ml). Cells were then collected at regular intervals. For TXY based ESC lines we have plated them at a density of 1mln/T75. After 24hrs of culture in DMEM/15%FCS+LIF cells were washed twice with PBS and medium was changed to DMEM/10%FCS-LIF with or without doxycycline (1.5ug/ml). Cells were collected after 48hrs of differentiation. For HDAC inhibition cells were pre-treated for 1hr with: 10 μM BRD668; 10 μM RGFP966; 10 μM Tasquinimod; 5 μM Tubastatin A; 5 μM PCI-34051; 30nM LMK235.

#### Native ChIP-seq

Cells were collected using Accutase (Thermo Fisher Scientific), washed twice in ice-cold PBS and counted. Typically, 3.5mln cells were used per immunoprecipitation (IP). A fraction of cells was always used for RNA/FISH verification of *Xist* induction. Cell pellet was resuspended in 90 μL (per 10 mln cells) of Lysis Buffer (50 mM Tris-HCl, pH7.5; 150mM NaCl; 0.1% sodium deoxycholate; 1% Triton X-100; 5mM CaCl_2_; Protease Inhibitor Cocktail; 5 mM sodium butyrate). After lysing cells on ice for 10 min we added 62 μL (per 10 mln cells) of Lysis Buffer with MNase (500 μL buffer + 0.5 μL MNase). Chromatin was digested for exactly 10 min at 37°C and reaction was stopped by the addition of 20 mM EGTA. To remove undigested debris the lysates were centrifuged at 13000 rpm for 5 min at 4°C. Supernatant was transferred to a fresh tube, an equal volume of STOP Buffer (50 mM Tris-HCl, pH7.5; 150mM NaCl; 0.1% sodium deoxycholate; 1% Triton X-100; 30 mM EGTA; 30 mM EDTA; Protease Inhibitor Cocktail; 5 mM sodium butyrate) was added, samples were stored on ice.

5 μL of lysate was digested in 45 μL of ProtK Digestion Buffer (20 mM HEPES; 1 mM EDTA; 0.5% SDS) for 30 min at 56°C. 50 μL of AMPure XP beads were added to the digested lysate together with 60 μL of 20% PEG8000 1.25M NaCl. After mixing the samples were incubated for 15 min at RT. Beads were separated on a magnet and washed twice with 80% Ethanol for 30sec. DNA was eluted in 12 μL of Low-EDTA TE and DNA concentration was measured using Qubit DNA High-Sensitivity kit. These measurements were used to normalize lysate concentration between samples. DNA isolated in this step was used as the input sample. The volume of each undigested lysate was adjusted for equal concentration and to obtain 1mL per IP using a 1:1 mix of Lysis Buffer and STOP Buffer.

Anti-mouse Dynabeads (50ul/IP) and Protein-A Dynabeads (10ul/IP) were washed twice in Blocking Buffer (0.5% BSA; 0.5% Tween in PBS). Beads were then resuspended in Blocking buffer and coated with antibodies for 4hrs at 4°C (anti-mouse Dynabeads: H3K9ac[1ug/IP], H3K4me3[2.5ug/IP], H3K27ac[1ug/IP]; Protein-A dynabeads: H3K4me1[0.4ug/IP], H3K27me3[1ug/IP], H2AK119Ub[0.4ug/IP], H4ac[2ug/IP]). Once coated beads were magnet-separated and resuspended in 1mL of concentration-adjusted lysate. Samples were left rotating overnight at 4°C.

Following day beads were magnet-separated and washed quickly with ice-cold washing buffers. anti-H3K4me3 IP was washed 8 times with Low Salt Buffer (0.1% SDS; 1% Triton X-100; 2 mM EDTA; 20 mM Tris-HCl, pH 8.1; 150 mM NaCl; 0.1% sodium deoxycholate). All remaining IPs were washed 4-times with Low Salt Buffer, 2-times with High Salt Buffer (0.1% SDS; 1% Triton X-100; 2 mM EDTA; 20 mM Tris-HCl, pH 8.1; 360 mM NaCl; 0.1% sodium deoxycholate) and 2-times with LiCl buffer (0.25 M LiCl; 1% NP40;1.1% sodium deoxycholate; 1 mM EDTA; 10 mM Tris-HCl pH 8.1). Prior to elution all samples were rinsed once in TE. ChIP-DNA was eluted in ProtK-Digestion buffer for 15 min at 56°C. Beads were separated and the supernatant was further digested for another 2 hr at 56°C. DNA was isolated using AMPure XP beads as described for the input sample.

For each nChIP-seq, 0.5 μL of each sample was used for qPCR validation of enrichment at control regions. 0.5 μL of input samples were also used to verify the digestion efficiency using D1000 tapestation. Remaining DNA concentration was adjusted and used for library preparation using Ovation^®^ Ultralow Library System V2 following suppliers protocol. Amplified libraries were size-selected for dinucleotide fraction (350-600bp fragments) using agarose gel-separation and MinElute Gel Extraction Kit (QIAGEN). Sample quality was inspected using D1000 tapestation. Samples were sequenced with HiSeq2500 using PE100 mode for nChIP-seq on TX1072 cell line and SE50 mode for nChIP-seq on TXY cell line.

#### EpiLC induction

2.5x10^5^ of ESC were plated onto fibronectin-coated 6-well in N2B27 medium (50%DMEM/F12 and 50% Neurobasal Medium supplemented with: 2-Mercaptoethanol[0.1mM], L-Glutamine[20mM]; B27 supplement[1x], Ndiff Neuro-2 Medium Supplement[1x]) with bFGF (12 ng/ml) and ActivinA (20 ng/ml) and with or without doxycycline (1ug/mL). Medium was changed every day and cells were collected at appropriate intervals.

#### RNA FISH

ESC were dissociated using Accutase (Invitrogen) and adsorbed onto Poly-L-Lysine (Sigma) coated coverslips #1.5 (1mm) for 5 min. Cells were fixed with 3% paraformaldehyde in PBS for 10 min at room temperature and permeabilised for 5 min on ice in PBS containing 0.5%Triton X-100 and 2mM Vanadyl- ribonucleoside complex (New England Biolabs). Coverslips were preserved in 70% EtOH at −20°C. Prior to FISH, samples were dehydrated through an ethanol series (80%, 95%,100% twice) and air-dried quickly. For detecting *Huwe1* and *Atrx*, a BAC spanning the respective genomic region (RP24-157H12, RP23-160I15) was labeled by nick translation (Roche) using spectrum green (Abbot). Per coverslip, 60ng probe was ethanol precipitated with Cot1 repeats, resuspended in formamide, denatured (10min 75°C) and competed for 45min at 37°C. For *Xist* detection or intron-spanning plasmid probe p510 was labeled by nick translation (Roche) using spectrum red (Abbot). Probe was ethanol precipitated and resuspended in formamide and denatured (10min 75oC). Both *Xist* and AtrX or Huwe1 probes were co- hybridized in FISH hybrization buffer (50% Formamide, 20% Dextran sulfate, 2x SSC, 1 μg/μl BSA, 10mM Vanadyl-ribonucleoside) over-night. Washes were carried out at 42°C three times 5min in 50% formamide in 2X SSC at pH = 7.2 and three times 5min in 2X SSC. 0.2mg/ml DAPI was used for counterstaining and mounting medium consisted of Vectashield (Vectorlabs). Images were acquired using an Inverted Confocal Spinning Disk Roper/Nikon.

#### IF with RNA FISH

Cells were grown on fibronectin coated coverslips #1.5 (1mm) and treated with doxycycline (1ug/mL) for 24hrs. Cells were fixed with 3% paraformaldehyde in PBS for 10 min at room temperature and permeabilised for 5 min on ice in PBS containing 0.5%Triton X-100 and 2mM Vanadyl- ribonucleoside complex (New England Biolabs). Samples were blocked for 15 min at room temperature with 1%BSA/PBS. Coverslips were incubated with primary antibodies diluted in blocking solution (anti-H4ac: 1/500; anti-H3K27ac:1/100; anti-H2AK119Ub: 1/200) in the presence of a Ribonuclease Inhibitor (0.8u/mL; Euromedex) for 45 min at room temperature. Coverslips were washed thrice with PBS for 5min and subsequently incubated with secondary antibody solution (goat anti-mouse or rabbit conjugated with Alexa fluorophores; 1/500; supplemented with Ribonuclease Inhibitor [0.8u/mL; Euromedex]) for 45 min at room temperature. Coverslips were washed three times with PBS for 5 min at room temperature. Cells were fixed again with 2% paraformaldehyde in PBS for 10 min at room temperature. After rinsing twice in 2xSSC coverslips were hybridized for *Xist* detection. Here we used a custom designed strand-specific probe that covers all exons with ∼75 bp long oligo nucleotides end-labeled with the Alexa 488 fluorophore (Roche). Probes was hybridized in FISH hybridization buffer (50% Formamide, 20% Dextran sulfate, 2x SSC, 1 μg/μl BSA, 10mM Vanadyl-ribonucleoside) over-night. Washes were carried out at 42°C three times 5min in 50% formamide in 2X SSC at pH = 7.2 and three times 5min in 2X SSC. 0.2mg/ml DAPI was used for counterstaining and mounting medium consisted of Vectashield (Vectorlabs). Images were acquired using an Inverted Confocal Spinning Disk Roper/Nikon. For signal quantification, signal intensity was measured along an axis across the *Xist* domain (z-plane with *Xist* maximum intensity) and part of the nucleus. Signal was not measured across the nucleoli. Profiles from 70-100 nuclei were centered at the point of *Xist* cloud boundary (maximum inflection point of *Xist* signal profile). Signal was normalized at per cell basis. In Figure 5B and Figure S5B shown are average profiles from all nucleoli with shading representing 25 and 75 quantiles.

#### RNA extraction, reverse transcription, pyrosequencing, qPCR

RNA was extracted according to the manufacturer’s recommendations using RNeasy Mini Kit (QIAGEN) with on-column DNase digestion (QIAGEN). For cDNA synthesis 1.1 μg RNA was reverse transcribed using Superscript III Reverse Transcriptase (Thermo Fisher Scientific). For allelic-skewing analysis the cDNA was PCR-amplified with biotinylated primers and sequenced using the Pyromark Q24 system (QIAGEN). To quantify ChIP DNA enrichments at control regions 2x SybRGreen Master Mix (Applied Biosystems) and a ViiA7 system (Applied biosystems) were used.

#### RNA-seq

RNA was prepared as described above and quality of samples were verified by Tapestation. Only samples with RIN score above 9 were processed. 100 ng of RNA was used for library preparation using Ovation Mouse RNA-Seq System that uses mouse-specific InDA-C depletion of rRNA. Manufacturers recommendations were followed. Libraries were sequenced using HiSeq2500 at PE100 settings.

#### TT-seq

The TT-seq protocol was performed as previously described with modifications (Schwalb et al., 2016). Cells were induced with DOX as described above in two T75 flasks per sample. After 0, 4, 8, 12 and 24 hr, RNA was labeled by the addition of 0.5 mM 4-thiouridine (4sU, Carbosynth) for 5 min. Medium was quickly removed and cells were lysed with 10 mL of TRI reagent (Sigma-Aldrich). Lysates were stored at −80°C.

The spike-in mix, containing three 4sU-labeled and three unlabelled RNAs, was synthesized *in vitro* with MEGAscript T7 Transcription Kit (Thermo Fisher), where 1/10 UTP in the transcription reactions was replaced with 4sUTP (Jena BioScience) for the 4sU-labeled spike-ins. RNA lysates were thawed on ice and 24 ng of spike-in mix was added per 10 million cells. The RNA was then purified according to the manufacturer’s protocol. 4sU-labeled RNA was isolated from 300 μg total RNA, for this two aliquots of 150 μg were sheared in Covaris MicroTubes with the Covaris S220 System (Covaris) for 10 s with settings 100 W and 1% duty cycle. These were then pooled and 2 μg of sonicated total RNA was taken for total RNA fraction. The RNA was biotinylated with EZ-Link HPDP-Biotin (Thermo Fisher) in two biotinylation reactions per sample (150 μg RNA in 10 mM Tris pH 7.5; 1 mM EDTA, 40% DMSO; and 200 μg/ml HPDP-biotin) that were incubated 1.5 h at 24°C. The biotinylated RNA was extracted with chloroform and precipitated with isopropanol. 4sU-labeled RNA was isolated by incubating the biotinylated RNA with paramagnetic streptavidin μMACS MicroBeads (Miltenyi) for 15 min at room temperature and binding to MACS columns. The columns were washed three times with wash buffer (100 mM Tris pH 7.5; 10 mM EDTA; 100 mM NaCl; and 0.1% Tween 20) at 65°C and three times with wash buffer at room temperature. The RNA was eluted from the columns with 100 mM DTT. The eluted 4sU-labeled RNA and reserved total RNA were purified with the miRNeasy Micro kit (QIAGEN) according to the manufacturer’s protocol with on-column DNase I treatment. 150 ng and 500 ng of isolated newly synthesized RNA and total RNA fractions, respectively, were used to prepare NGS libraries with the TruSeq Stranded Total RNA Library Prep Kit (Illumina). The sample libraries were pooled and sequenced as a single run on the Nextseq550 (Illumina) using PE150 mode.

#### CRISPR/Cas9 knockout of *Hdac3*

Two gRNAs have been designed as previously described (Haeussler et al., 2016) and cloned into pX462 vector allowing for their expression under U6 promoter. Additionally, this vector leads to expression of Cas9. TX1072 ESCs were transfected using AmaxaTM 4D-NucleofectorTM system (Lonza) as per manufacturer’s protocol. After a pulse of puromycin selection cells were plated at clonal density. Single clones were picked, expanded and screened for deletion by PCR. Final two knockout clones were further validated by sequencing both alleles. *Hdac3*^*−/−*^*#1* harbors a deletion of exon 4-7 on the Cast allele and exon 9 on the B6 allele. *Hdac3*^*−/−*^*#2* harbors a deletion of exon 4-7 on the B6 allele and exon 7 on the B6 allele. Both mutants showed negligible expression on the mRNA level as well as no detectable protein when using a N terminus specific antibody (Figure 4B).

#### CRISPR/Cas9 FLAG tagging of *Hdac3*

The *Hdac3-Aid-3xFlag* targeting construct was generated as follows: 500bp homology arms (flanking both sides of, but excluding the stop codon of *Hdac3*) were PCR amplified from mouse genomic DNA. 1-step Gibson cloning (NEB) was subsequently used to simultaneously surround the digested AID-3xFLAG insert (carrying a puromycin resistance gene under the control of the PGK promoter) in frame with the homology arms and clone the insert into the pBR322 vector. Synonymous mutations in the PAM/SEED target sequence (located on the 5′ homology arm) were then introduced using the QuickChange II XL site-directed mutagenesis kit (Agilent) to prevent Cas9 mediated cleavage of the targeting vector upon transfection and of the 3xFLAG tagged allele(s) upon integration.

Single gRNAs has been designed as previously described (Haeussler et al., 2016) and cloned into pX462 vector allowing for its expression under U6 promoter.

Hdac3 targeting was performed using the 4-D nucleofector system from Lonza. 5 million cells were electroporated with 2.5 μg each of non-linearized targeting vector and gRNA/Cas9 encoding plasmids. The day after, puromycin was added at a concentration of 0.4ug/mL. Single clones were picked, expanded and screened for insertion by PCR. One clone showing homozygous insertion of the FLAG tagged cassette at the *Hdac3* locus was validated by sanger sequencing and western blotting and kept for subsequent experiments (Figure S5).

#### HDAC3-FLAG IP-Mass Spectrometry

50 mln (per IP) ESCs (TX1072 –DOX; *Hdac3*^*Flag/Flag*^ +DOX 24h) were trypsinised and washed in PBS. Pellets were snap frozen and stored at −80°C. Defrosted pellets were resuspended in 10 mL of buffer A (10 mM HEPES pH 7.9; 5 mM MgCl_2_; 10 mM KCl; 1 mM DTT; 0.1% NP-40, protease inhibitors) and incubated for 10 min at 4°C. Nuclei were pelleted at 2000 rpm at 4°C for 10 minutes. Nuclei were resuspended in 500μL of buffer C (20mM HEPES pH 7.9; 20% (v/v) glycerol; 150 mM KCl; 5 mM MgCl2; 2 mM EDTA; 2 mM EDTA; 1 mM DTT; protease inhibitors) and sonicated 3 times for 10 s (Bioruptor, medium setting). Lysates were cleared by centrifugation 12000 rpm at 4°C for 20 minutes. Lysate concentrations were equalised based on protein concentration. Anti-FLAG M2 Magnetic Beads (Sigma-Aldrich) were washed twice in buffer C and then added to each lysate (50μL per IP). Lysates were left rotating over-night at 4°C. Next day beads were washed 5 times in wash buffer (20 mM HEPES pH 7.9; 0.1% NP-40; 150mM KCl; 5mM MgCl2; 2mM EDTA; 2 mM EDTA; 1 mM DTT).

Proteins on magnetic beads were washed twice with 100 μL of 25mM NH4HCO3 and on-beads digestion was performed with 0.2 μg of trypsin/LysC (Promega) for 1 hour in 100μL of 25mM NH4HCO3. Samples were then loaded onto a custom-made C18 StageTips for desalting. Peptides were eluted using 40/60 MeCN/H2O + 0.1% formic acid and vacuum concentrated to dryness.

Online chromatography was performed with an RSLCnano system (Ultimate 3000, Thermo Scientific) coupled online to an Orbitrap Fusion Tribrid mass spectrometer (Thermo Scientific). Peptides were trapped on a C18 column (75 μm inner diameter × 2 cm; nanoViper Acclaim PepMap 100, Thermo Scientific) with buffer A (2/98 MeCN/H2O in 0.1% formic acid) at a flow rate of 4.0μL/min over 4 min. Separation was performed on a 50cm x 75 μm C18 column (nanoViper Acclaim PepMap RSLC, 2 μm, 100Å, Thermo Scientific) regulated to a temperature of 55°C with a linear gradient of 5% to 25% buffer B (100% MeCN in 0.1% formic acid) at a flow rate of 300 nL/min over 100 min. Full-scan MS was acquired in the Orbitrap analyzer with a resolution set to 120,000 and ions from each full scan were HCD fragmented and analyzed in the linear ion trap.

#### Anti-FLAG ChIP-seq

Cells were grown in 145mm plate format. Cells were initially fixed in 1.5 mM ethylene glycolbis [succinimidyl succinate] (EGS) for 30 min, next 2% formaldehyde was added and further incubated for 10 min. After rinsing twice in PBS, cells were scraped and pellets directly used for ChIP.

Fixed pellets were lysed in 6 mL Nuclear Lysis Buffer (0.5% Triton X-100, 0.1 M sucrose, 5 mM MgCl2, 1 mM EDTA, 10 mM Tris-HCl pH 8.0, 1x Protease Inhibitors) for 10min on ice and dounce-homogenized. Nuclei were pelleted and resuspended in 0.9 mL Lysis Buffer (1 mM EDTA, 0.5 mM EGTA, 10 mM Tris pH 8.0, 0.5% N-Lauroylsarcosine, 1x Protease Inhibitors). Chromatin was sheared using a BioruptorPlus (Diagenode) set to high for 30 cycles. Unsonicated chromatin was removed by centrifugation. Supernatant was diluted with 3.6 mL of Dilution Buffer (1.25% Triton, 0.125% sodium deoxycholate, 6 mM EDTA, 10 mM Tris-HCL pH8.0, 1x Protease Inhibitors) and antibody coated beads were added (per IP: 10 μL of anti-FLAG antibody). Samples were left rotating overnight at 4°C.

Following day beads were magnet-separated and washed 5-times with LiCl buffer (0.50 M LiCl; 1% NP40; 1.1% sodium deoxycholate; 1 mM EDTA; 10 mM Tris-HCl pH 8.1) and 5 times with High Salt Buffer (0.1% SDS; 1% Triton X-100; 2 mM EDTA; 20 mM Tris-HCl, pH 8.1; 1M NaCl; 0.1% sodium deoxycholate). Each wash was performed for 10 min on a rotating wheel at 4°C. Prior to elution all samples were rinsed once in TE. ChIP-DNA was eluted in ProtK-Digestion Buffer (20mM HEPES; 1mM EDTA; 0.5% SDS; 0.8mg/mL Proteinase K) for 15min at 56°C. Beads were separated and the supernatant was further digested for another 2hrs at 56°C and then decrosslinked for 4hrs at 68°C. DNA was isolated using AMPure XP beads. Libraries were prepared using Ovation Ultralow Library System V2 following suppliers protocol. Samples were sequenced with HiSeq2500 using PE100 mode.

### Quantification and Statistical Analysis

#### Time course analysis on TX1072 cell line

##### nChIP-seq Data processing

Adapters and low quality bases (< Q20) have been removed with TrimGalore (v0.4.0; http://www.bioinformatics.babraham.ac.uk/projects/trim_galore) and Cutadapt (1.8.2) (Martin, 2011). An ‘N-masked” genome has been generated with SNPSplit (0.3.2) (Krueger and Andrews, 2016) which is a version of the mouse reference genome mm10 where all the polymorphic sites for the hybrid strain *Mus musculus* CAST/EiJ and *Mus musculus* C57BL/6 are masked by ambiguity nucleobase ‘N’. For all samples, reads were then mapped to the ‘N-masked” genome with Bowtie2 (2.2.5) with options [--end-to-end -N1 -q] (Langmead and Salzberg, 2012). Duplicates were discarded with Picard MarkDuplicates (1.65) with options [REMOVE_DUPLICATES = true] (https://broadinstitute.github.io/picard/) and reads mapped on blacklisted regions from Encode Consortium were discarded. SNPSplit (0.3.2) (Krueger and Andrews, 2016) was then used to generate allele-specific BAM files by separating the alignment into two distinct alleles (CAST and B6) based on SNPs information downloaded from Sanger. Bigwig files were created with bedtools genomeCoverageBed (2.25.0) (Quinlan and Hall, 2010), using a scale factor calculated on the total library (10.000.000/total reads) for both allele specific bigwigs, and loaded on UCSC genome browser.

#### Analysis of active histone marks (H3K27ac, H4ac, H3K9ac, H3K4me3, H3K4me1)

##### Peak identification

Peak calling was done with MACS2 (2.0.10) (Zhang et al., 2008) with options [--broad -B -f BAMPE --broad-cutoff 0.01], on total (non allele-specific) ChIP-seq signal with input as control. Then, peaks with a fold change inferior to 3 were filtered out. For each histone mark, consensus peaks were defined as follow. For each replicate, all peaks coordinates were merged using bedtools merge (2.25.0) (Quinlan and Hall, 2010). Then, common regions between merged peaks coordinates of each replicate were selected using bedtools intersectBed (2.25.0) (Quinlan and Hall, 2010).

##### Counts and normalization

Total and allelic reads overlapping consensus peaks were counted using featureCounts (1.5.1), with options [ -C -p -P] (Liao et al., 2014). Only peaks with more than 50 total allelic reads were selected. Next, ratios of allelic counts, called d-scores [reads^B6^/(reads^Cast^+ reads^B6^)], were calculated.

##### Analysis of the dynamics

The dynamic evolution of d-score with time was analyzed for each peak with a d-score calculated on the 10 samples (5 times, 2 replicates). Moreover, only peaks that had a biallelic d-score at time 0 (comprised between 0.3 and 0.7) were selected. Then normalized d-scores were calculated by dividing each d-score by the corresponding initial d-score (time 0) and by 2, so that initial normalized d-scores start at 0.5. Initially we have fitted either exponential or sigmoidal curve to these values. We compared the quality of this fitting by taking into account the number of parameters used in the models (AIC and BIC criteria) and concluded that the sigmoidal model best reflects the observed dynamics (data not shown). Sigmoidal fitting of normalized d-scores in function of time has been done for each peak with a four-parameter log-logistic function from drc R package (Ritz et al., 2015). Sigmoidal fittings with low residuals (< 0.2; corresponding to more than 98% of the peaks) were selected, and three parameters were obtained from these fittings: IC35, which is the time where the normalized d-score reaches 0.35 on the sigmoidal fitting; ED_50_, which corresponds to the maximum slope of the sigmoidal fitting; and the minimum second derivative used as a proxy for when the curve starts to decrease. The minimum of the second derivative has been calculated using optimize function from R in the interval between 0 and the calculated ED_50_ to avoid finding a local minimum not corresponding to the real minimum. IC/ED/minimum second derivative superior to 24h (outside the time range of the experiment) or not calculated were considered windows with a late loss of active histone mark, and replaced by 24h.

Moreover, for all active histone marks, IC25, IC30 and IC40 (thresholds of normalized d-score:0.25; 0.3; 0.4) were also tested and were able to replicate all our major findings (data not shown).

##### Features comparison between early and late silenced peaks

Early, intermediate and late silenced peaks located on TSS were defined with a 3 clusters k-means based on IC35 values. Early and late silenced peaks were compared for several features using Wilcoxon test: level of expression of associated genes (from TT-seq - see below), distance to *Xist*, distance to the closest TAD boundary (Dixon et al., 2012), distance to the closest LAD (Peric-Hupkes et al., 2010), LINE density in a window of 100kb around the peak (from RepeatMasker database) and gene density in a window of 100kb around the peak. Moreover, IC35 was compared for peaks that are inside or close to *Xist* entry sites (Engreitz et al., 2013)(< 100kb) and those that are distant.

For comparison of dynamics (Figure 6E) of deacetylation and accumulation of repressive marks, early, mid-early, mid-late and late silenced genes were defined with a 4 clusters k-means based on IC35 values.

##### H3K4me1 accumulation at TSS

Average plots of H3K4me1 signal were created using DeepTools (3.0.2) (Ramírez et al., 2014). Matrix counts were created using DeepTools computeMatrix around active TSS (see above) on chrX and autosomes separately (with option [--binSize 50]), plots were then created using DeepTools PlotProfile.

#### Analysis of repressive marks (H3K27me3, H2AK119Ub)

##### Windows definition

For repressive marks, global analysis was first done on fixed windows (10 kb) spanning the whole genome, then on different genomic subcategories: active gene bodies, active promoters, active enhancers and intergenic regions. Active genes were defined as genes with a transcript having its TSS (refFlat annotation) overlapping a consensus peak of H3K9ac and a consensus peak of H3K4me3. For genes having several active transcripts detected, the active gene was defined as starting at the minimum start of transcripts, and ending at the maximum end of transcripts. Then, the active gene bodies were defined as those active genes excluding the 2 first kb downstream of TSS. Active promoters were defined as ± 2kb windows around the TSS of active genes. Putative, active enhancers were defined as intersect between consensus H3K27ac peaks and consensus H3K4me1 peaks, located at a minimal distance of 1kb from a TSS. Intergenic regions were defined as 10 kb windows not overlapping a gene (active or inactive) and its promoter (2kb downstream) or an active enhancer. Enhancer coordinates were also used for the analysis of active histone mark (H3K4me1 and H3K27ac).

##### Counts and normalization

It should be noted that a d-score analysis, as used for active mark loss, involves the use of the active allele as an internal control, but in the case of H2AK119Ub and H3K27me3, this was hindered by very low enrichment for repressive marks across the Xa. For all defined windows, total and allelic reads overlapping those features were then counted using featureCounts (1.5.1), with options [-C -p -P] (Liao et al., 2014). Then, analysis was done based on normalized reads from B6 allele (allele of the inactive X chromosome). For each sample, a normalization factor was calculated with the trimmed mean of M-values method (TMM) from edgeR package (Robinson et al., 2010), based on B6 reads overlapping consensus peaks located on autosomes (identified and defined as consensus such as for active histone marks analysis). To correct for chromatin accessibility or mappability bias, 10kb windows with outliers counts in the input (counts superior or inferior to mean +-1.5 sd) were discarded from the analysis. Moreover, to represent B6 read accumulation compared to time 0, subtraction of normalized initial counts (time 0) was then applied to all other time points.

##### Analysis of the dynamics

Sigmoidal fitting of B6 read accumulation in function of time has also been done with the four-parameter log-logistic function from drc R package. Sigmoidal fittings with low residuals (< mean(residuals) + 1.5 sd(residuals)) were selected, representing more than 90% of the analyzed windows. The ED_50_ and the maximum second derivative of the sigmoidal fitting were calculated for each window. Windows with ED_50_/maximum 2nd derivative superior to 24h, or not calculated, were considered as windows with a late accumulation of repressive marks, those were then replaced by 24h. For pairwise comparison between marks, only windows with ED_50_/maximum 2nd derivative inferior to 24h for both marks were selected. For comparison of ED_50_ between different genomic subcategories (active promoters, gene bodies, enhancers, intergenic regions) for a same histone mark, no filter was done.

To test the stability of our results, main analysis was also performed with different thresholds of residuals (0.2; 0.15; 0.1; 0.05). Moreover, repressive marks dynamics were also analyzed using allelic ratio (d-scores), such as for analysis of active histone marks, using ED_50_, IC60 and IC65 as criteria. All those analysis revealed stable and reproducible results (data not shown).

#### TT-seq

Adapters (“TruSeq Universal Adapter”) and low quality bases (< Q20) have been removed with Cutadapt (v1.16) (Martin, 2011). For all samples, reads were then mapped to the ‘N-masked’ genome (masked for SNPs of the hybrid strain *Mus musculus* CAST/EiJ and *Mus musculus* C57BL/6) with STAR 2.3.0 (Dobin and Gingeras, 2015) with maximum 2 percent mismatches. Only unique alignments were retained and soft clipping was disabled [mapping parameters: ‘--outFilterMismatchNoverReadLmax 0.02 --outFilterMultimapScoreRange 0 --alignEndsType EndToEnd’]. Samtools (Li et al., 2009) was used to quality filter SAM files, where alignments with MAPQ smaller than 7 (-q 7) were skipped and only proper pairs (-f 2) were selected. SNPSplit (v0.3.2) (Krueger and Andrews, 2016) was used to generate allele-specific SAM files. Then, total and allelic reads per gene were counted with featureCounts (1.5.1) [options: -C -p -s 2 -T 8] (Liao et al., 2014) on longest gene coordinates (refFlat annotation). The next steps of analysis (from d-scores to sigmoidal fitting) were done such as for nChIP-seq analysis, except that d-scores were calculated for genes with minimum 10 allelic reads instead of 50. Total normalized expression used for features comparison of early and late silenced peaks (see upper) was calculated based on signal in introns and exons, normalized by the size of the library and the size of the entire gene.

#### Analysis of WT and Hdac3^−/−^

##### RNA-seq

Reads were first mapped on rRNA with Tophat (2.1.0) (Trapnell et al., 2009), with options [-g 1 --no-coverage-search --library-type fr-secondstrand]. Paired unmapped reads were then used to reconstruct fastq files with bedTools bamToFastq (2.25.0) (Quinlan and Hall, 2010). Those files were then mapped with Tophat (2.1.0) (Trapnell et al., 2009), with options [-p 8 -g 1 -x 1 -N 3 --read-edit-dist 3 --no-coverage-search --library-type fr-secondstrand], with refFlat annotation. Reads covering exons of each gene were then counted with featureCounts (1.5.1) with options [-C -p -s 1 -T 8] (Liao et al., 2014). D-scores were then calculated for genes with minimum 10 allelic reads.

For differential gene expression analysis prior to DOX treatment, analysis was done on genes for which 2 among the 4 samples have a TPM superior to 1. Normalization was done using TMM (Robinson et al., 2010) method from edgeR and differential analysis using Voom function from Limma R package (Ritchie et al., 2015).

##### nChIP-seq

The processing from raw data (fastq) to clean mapped file was done such as for time course nChIP-seq. Then, for active histone marks, the reads counts of H3K27ac and H4ac nChIP-seq were done on consensus H3K27ac peaks and consensus H4ac peaks defined in time course analysis, using featureCounts (1.5.1), with options [-C -p -P] (Liao et al., 2014). D-score was calculated with a slight modification for one of the 2 mutant sample. Indeed, this sample showed a non biallelic signal on X chromosome in its input, due to the presence of some XO cells. To take this bias in account, a normalization factor was calculated as follow: normFactor = (reads^B6^/(reads^B6^ + reads^CAST^)) / (1-(reads^B6^/(reads^B6^ + reads^CAST^))).

Then d-score for this mutant was adjusted from classic d-score as follow: d-score = d-score/(d-score + normFactor - normFactor ^∗^ d-score). After this adjustment, only the peaks that were biallelic at time 0 in WT and mutant samples were selected (d-scores between 0.3 and 0.7) for analysis. For repressive marks, analysis was done such as for time course nChIP-seq, based on signal accumulation of 10 kb windows all along the X chromosome.

For differential peak signal analysis at t = 0hr, normalization was done using TMM (Robinson et al., 2010) method from edgeR and differential analysis using Voom function from Limma R package (Ritchie et al., 2015).

##### Comparison of sensitive and resistant peaks (nChIP-seq on active histone marks) / genes (RNA-seq, ChIP-seq on repressive histone marks)

Sensitive and resistant peaks/genes were defined as follow. For nChIP-seq on active histone marks and RNA-seq, only peaks/genes with d-scores considered as stable between replicates (diff replicate < median + 2sd of all differences between replicates) for both clones (WT, mutant) at each time point were selected. D-scores were normalized to time 0 (d-score / d-score t0), then only peaks/genes considered as not escaping were selected (normalized d-score at t24 < 0.8). Difference of normalized d-score at 24h between WT and mutant was then calculated, and divided by the difference of normalized WT d-score between 0 and 24h for the WT sample. K-means with 3 clusters was done on this normalized difference, identifying 3 groups: sensitive, intermediate and resistant peaks/genes. For nChIP-seq on repressive histone marks, differential enrichment between WT and mutant was defined as the difference between WT normalized BL6 enrichment relative to time 0 and Mutant normalized BL6 enrichment relative to time 0, divided by WT normalized BL6 enrichment relative to time 0.

#### Analysis of FLAG ChIP-seq on Hdac3^Flag/Flag^ ESC cell line

The processing from raw data (fastq) to d-score calculation was done such as for time course nChIP-seq, with slight modifications. The peak calling was done with MACS2 (2.0.10) (Zhang et al., 2008) with options [-B -f BAMPE], on total (non allele-specific) ChIP-seq signal against the FLAG ChIP-seq on TX1072 cell line as control. Consensus peaks were defined as peak regions present at least in 2 of the 4 samples, using bedtools multiIntersectBed (2.25.0) (Quinlan and Hall, 2010). Only peaks with more than 20 total allelic reads were selected.

Heatmaps and average plots of HDAC3 signal in comparison with H3K27ac and H4ac signal were created using DeepTools (3.0.2) (Ramírez et al., 2014). Merged peaks of H3K27ac and H4ac at time 0 were split in 2 categories: active promoters for merged peaks overlapping a TSS and putative active enhancer for merged peaks at a distance of minimum 2 kb of a TSS. The merged peaks were ordered by normalized signal of HDAC3 peaks (signal/length of the peak) overlapping those peaks. For HDAC3, signal of the control (FLAG ChIP-seq on TX1072 cell line) was subtracted from the signal of FLAG ChIP-seq on *Hdac3*^*Flag/Flag*^ ESC cell line using DeepTools bamCompare with options [--ratio subtract --binSize 100] before to proceed to the next step. Then, matrix counts were created using DeepTools computeMatrix around TSS of active promoters (with option [--binSize 100]) or centers of putative enhancers, plots were then created using DeepTools PlotHeatmap.

For H3K27ac and HDAC3 peaks annotation comparison (Figure S5C), peaks were annotated as follow: peaks at a distance < 2000 bp of a TSS were considered in promoters, other peaks were considered as intragenic if overlapping a gene body, intergenic if not.

Category of peaks accumulating signal or not on Xi (Figures S5E and S5F) were created based on clustering with hclust function with parameter [method = ’Ward.D’], using Pearson correlation as distance. 11 clusters were identified using cutree function. Those clusters were grouped in 2 super groups according to the presence or not of an accumulation of signal on Xi at 4hr in each cluster.

#### Analysis of TXY:Xist and TXY:XistΔA

##### nChIP-seq

Processing steps to clean bam files were similar to time course nChIP-seq analysis, except that mapping was not allele-specific but done on mm10 genome and that reads mapped with low quality (< q10) were removed with samtools (1.3)(Li et al., 2009). Such as for time course nChIP-seq analysis, ChIP-seq signal was analyzed differently depending on the histone mark: per peak for active mark (H3K27ac) and per window for repressive marks (H3K27me3, H2AK119Ub). Peak calling was done similarly to time course nChIP-seq analysis of active histone marks. Here, consensus peaks were defined as common regions between peaks identified in minimum 2 among the 4 samples using bedtools multiIntersectBed (2.25.0) and bedtools merge (2.25.0).

The windows for repressive mark analysis were defined similarly as for nChIP-seq time course analysis for repressive marks, except that active TSS were defined here based on consensus peaks of H3K27ac overlapping TSS, and putative active enhancers as H3K27ac peaks located at minimum 1kb of TSS.

Reads overlapping defined peaks (for active marks) or windows (for repressive marks) were then counted with featureCounts (1.5.1) (Liao et al., 2014) with default options.

For global analysis on repressive marks, counts normalization was done such as for time course nChIP-seq analysis, based on counts falling in autosomal consensus peaks. For windows and peaks analysis, windows that had less than one read per 50kb for more than 2 among the 8 samples were removed for the analysis. Normalization factors were calculated based on windows located on autosomes (with selection of the 10000 most higher signal for intergenic window analysis), with TMM method using edgeR (Robinson et al., 2010).

Because of the high variability in proportion of cells involved in X chromosome inactivation quantified by the presence of *Xist* Cloud by FISH experiments (TXY:*XistWT*#1 46.64%, TXY:*XistWT*#2 59.44%, TXY:*XistΔA*#1 50.61%, TXY:*XistΔA*#2 48.24%), linear regression including the percentage of induction calculated by FISH was fitted for each window according the following model: ∼0 + clones + clones: induction, using Voom function from Limma R package (Ritchie et al., 2015). The slope of this regression represents then the logFC between noDox and Dox conditions if the induction of the cell population was complete (corrected logFC).

Metaplots were created using DeepTools (3.0.2) (Ramírez et al., 2014). Bigwigs of log2(FC) between Dox and noDox samples were created with personalized scaling according to normalized factors calculated above using DeepTools bamCompare, bigwig of mean of log2(FC) between replicates was then created using DeepTools bigwigCompare (binSize: 100 bp for H3K27ac, 1000 bp for H3K27me3 and H2Ub), matrix counts was then created using DeepTools computeMatrix on active genes coordinates (see above) on chrX and autosomes separately and plots were then created using DeepTools PlotProfile.

#### Proteomics data analysis

For identification, the data were searched against the *Mus musculus (UP000000589) UniProt* database using Sequest HF through proteome discoverer (version 2.2). Enzyme specificity was set to trypsin and a maximum of two-missed cleavage sites were allowed. Oxidized methionine, N-terminal acetylation, and carbamidomethyl cysteine were set as variable modifications. Maximum allowed mass deviation was set to 10 ppm for monoisotopic precursor ions and 0.6 Da for MS/MS peaks.

The resulting files were further processed using myProMS v3.6 (Poullet et al., 2007). FDR calculation used Percolator and was set to 1% at the peptide level for the whole study. The label free quantification was performed by peptide Extracted Ion Chromatograms (XICs) computed with MassChroQ version 2.2.2 (Valot et al., 2011). For protein quantification, XICs from proteotypic peptides shared between compared conditions (TopN matching) with no missed cleavages were used. Median and scale normalization was applied on the total signal to correct the XICs for each biological replicate. To estimate the significance of the change in protein abundance, a linear model (adjusted on peptides and biological replicates) was performed and *p-value*s were adjusted with a Benjamini–Hochberg FDR procedure with a control threshold set to 0.05.

### Data and Software Availability

The accession number for the sequencing datasets reported in this paper is GEO: GSE116480. The mass spectrometry proteomics data have been deposited to the ProteomeXchange Consortium via the PRIDE partner repository with the dataset identifier PXD011344

## Supplemental Information

Table S1. List of Differentially Expressed Genes and GO Term Enrichment Analysis, Related to Figures 3 and S3

Table S2. List of Proteins Significantly Enriched in HDAC3-FLAG +DOX IP-MS Compared with No FLAG Line, Related to Figure 5

Table S3. List of DNA Oligonucleotides, Related to STAR Methods

Table S4. Sequence of TT-Seq Spike-Ins, Related to STAR Methods
